# Evaluation of two electronic-rehabilitation programmes for persistent knee pain: protocol for a randomised feasibility trial

**DOI:** 10.1136/bmjopen-2022-063608

**Published:** 2022-06-03

**Authors:** Dawn Groves-Williams, Gretl A McHugh, Kim L Bennell, Christine Comer, Elizabeth M A Hensor, Mark Conner, Rachel K Nelligan, Rana S Hinman, Sarah R Kingsbury, Philip G Conaghan

**Affiliations:** 1 Leeds Institute of Rheumatic and Musculoskeletal Medicine, University of Leeds, Leeds, West Yorkshire, UK; 2 School of Healthcare, University of Leeds, Leeds, West Yorkshire, UK; 3 Department of Physiotherapy, The University of Melbourne Centre for Health Exercise and Sports Medicine, Melbourne, Victoria, Australia; 4 Musculoskeletal and Rehabilitation Service, Leeds Community Healthcare NHS Trust, Leeds, West Yorkshire, UK; 5 NIHR Leeds Biomedical Research Centre, Leeds, West Yorkshire, UK; 6 School of Psychology, University of Leeds, Leeds, West Yorkshire, UK

**Keywords:** PAIN MANAGEMENT, Rheumatology, World Wide Web technology, Telemedicine

## Abstract

**Introduction:**

Persistent, knee pain is a common cause of disability. Education and exercise treatment are advocated in all clinical guidelines; however, the increasing prevalence of persistent knee pain presents challenges for health services regarding appropriate and scalable delivery of these treatments. Digital technologies may help address this, and this trial will evaluate the feasibility and acceptability of two electronic-rehabilitation interventions: ‘My Knee UK’ and ‘Group E-Rehab’.

**Methods and analysis:**

This protocol describes a non-blinded, randomised feasibility trial with three parallel groups. The trial aims to recruit 90 participants (45 years or older) with a history of persistent knee pain consistent with a clinical diagnosis of knee osteoarthritis. Participants will be randomly assigned in a 1:1:1 allocation ratio. The ‘My Knee UK’ intervention arm will receive a self-directed unsupervised internet-based home exercise programme plus short message service support (targeting exercise behaviour change) for 12 weeks; the ‘Group E-Rehab’ intervention arm will receive group-based physiotherapist-prescribed home exercises delivered via videoconferencing accompanied by internet-interactive educational sessions for 12 weeks; the control arm will receive usual physiotherapy care or continue with their usual self-management (depending on their recruitment path). Feasibility variables, patient-reported outcomes and clinical findings measured at baseline, 3 and 9 months will be assessed and integrated with qualitative interview data from a subset of Group E-Rehab and My Knee UK participants. If considered feasible and acceptable, a definitive randomised controlled trial can be conducted to investigate the clinical effectiveness and cost-effectiveness of one or both interventions with a view to implementation in routine care.

**Ethics and dissemination:**

The trial was approved by the West of Scotland Research Ethics Committee 5 (Reference: 20/WS/0006). The results of the study will be disseminated to study participants, the study grant funder and will be submitted for publication in peer-reviewed journals.

**Trial registration number:**

ISRCTN15564385.

**Strengths and limitations of this study**

- This study does not require any face-to-face contact as the interventions are accessed and/or delivered remotely.
- The digital health interventions being investigated have been tailored to the needs of individuals with persistent knee pain based on feedback from patient and public involvement and expert review groups conducted during phase I.
- Data will be collected and analysed using a mixed methods approach to provide a greater understanding of the feasibility and acceptability of the two digital health interventions.
- Participants, physiotherapists and the trials staff administering the study cannot be blinded; however, the trial statistician will remain blinded to group allocation until the database has been locked.
- The interventions require participants to have an active email account, mobile phone and access to a computer or tablet with Internet access suitable for receiving/making video calls.

## Introduction

Persistent or chronic knee pain, often with associated stiffness and functional limitations, is a common problem in older and middle-aged adults.^1 2^ A leading cause is osteoarthritis (OA) and in 2020, the pooled global prevalence of knee OA in individuals aged 40 and over was reported to be around 23%, with a positive correlation between prevalence and increased age.^3^ Approximately 10% of adults in the UK have a clinical diagnosis of OA, with symptomatic knee OA being the most common site.^4^ It is estimated that by 2035, the number of people with knee OA in the UK could reach 8.3 million.^5^ Exercise and access to appropriate education are advocated in all clinical guidelines as core treatments for persistent knee pain. However, with the increasing prevalence of persistent knee pain and current treatment delivery strategies not always addressing patient needs, managing these patients is challenging.^6^

Physiotherapists are key in providing education, exercises and self-management support to improve symptoms and function for individuals with knee OA.^7^ It has previously been observed that physiotherapists can effectively deliver OA rehabilitation interventions remotely using video technologies,^8–10^ which is increasingly important given the adoption of telehealth during the COVID-19 pandemic.^11–13^ It has been reported that since the onset of COVID-19, 88% of 50–64 year olds, 75% of 65–74 year olds and 46% of those aged 75 or over use the internet almost every day.^14^ These data support the assumption that some, but not all older individuals, could access internet-based electronic interventions.

Digital health interventions for knee pain have been developed and delivered by physiotherapists in Australia; and there is evidence to suggest they can improve knee pain and function^9 15^ and are generally accepted by patients with knee OA.^16^ Such digital health interventions may be useful in other large health services (such as in the UK) as an alternative to ‘in person’ clinic appointments, particularly where physiotherapy services are overstretched^17^ and waiting times are prolonged due to the COVID-19 pandemic,^18^ or where individuals are unable to attend physiotherapy sessions due to mobility problems and/or lack of transport.^19–21^

### Aims and objectives

This trial aims to evaluate the feasibility and acceptability of two different electronic-rehabilitation (e-rehabilitation) interventions in individuals with persistent knee pain. One comprises a self-directed internet-based home exercise programme with exercise behaviour change support provided via automated short message service (SMS) (‘My Knee UK’). The second is a group-based home exercise programme with internet-interactive education sessions (‘Group E-Rehab’), where the home exercise programme is prescribed and monitored by a physiotherapist using videoconferencing. The trial will also explore the effect of each e-rehabilitation programme on pain and other symptoms compared with the control arm and provide a report on any additional resources (eg, additional National Health Service (NHS)/private healthcare services) used by participants during the trial.

## Methods

### Trial design

**Figure 1:**
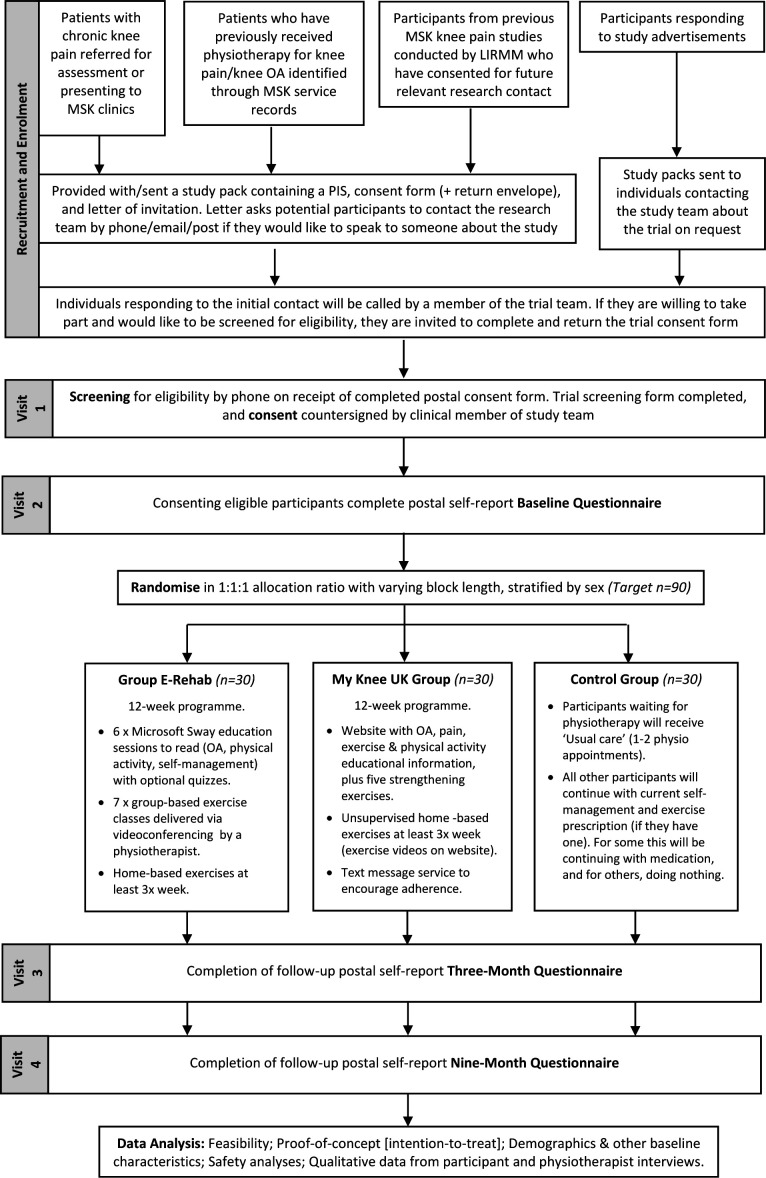
Study flow diagram. LIRMM, Leeds Institute of Rheumatic and Musculoskeletal Medicine; MSK, musculoskeletal; OA, osteoarthritis; PIS, Participant Information Sheet.

This is an unblinded, single-centre, randomised feasibility trial with three parallel arms (two e-rehabilitation treatment arms and one control arm). The study protocol was designed to conform to the Standard Protocol Items: Recommendations for Interventional Trials guidelines^22 23^ and the extension of the Consolidated Standards of Reporting Trials (CONSORT) statement for randomised pilot and feasibility trials.^24^ The feasibility trial (phase II) opened to recruitment in March 2021 and the planned study end date is June 2023. Trial phases are outlined in figure 1.

In line with the recently published CONSERVE 2021 Statement and guidelines,^25^ several protocol changes were made in response to the COVID-19 pandemic (online supplemental 1).

10.1136/bmjopen-2022-063608.supp1 Supplementary data

### Patient and public involvement

Members of the National Institute for Health Research Leeds Biomedical Research Centre Patient and Public Involvement (PPI) group provided input into the trial design and outcome measures through focus group discussions during the funding application stages. PPI was subsequently used to provide input into the refinement of the two e-rehabilitation programmes during the first phase of the study. Additionally, two PPI representatives are members of the project advisory group and help review content and assist with key decisions throughout the trial.

### Participants

The target population is adults with persistent knee pain who meet the eligibility criteria (table 1). Potential participants have either been referred to musculoskeletal (MSK) services for assessment or have previously received physiotherapy for persistent knee pain; are participants from past studies that have consented to future contact; have responded to a media campaign advertising for volunteers (figure 1).

**Table 1:** Trial eligibility criteria

Inclusion criteria	Exclusion criteria
Adults≥45 years Knee pain>3 months and on most days of previous month Knee pain during walking≥4 on an 11-point numerical rating scale^45^ Activity-related knee joint pain Has a mobile phone, active email account and computer with internet access suitable for receiving and making video calls if required	Inflammatory arthritis (including gout) A joint replacement in the study knee An injection into the study knee joint within the last month An arthroscopy of the study knee joint in the last 3 months Enrolled in another research study involving an intervention for osteoarthritis treatment or management Unable to comply with the study protocol Unable to understand written and spoken English

### Digital health intervention treatment arms

The e-rehabilitation interventions were adapted for use in the UK (My Knee UK) or developed (Group E-Rehab) during phase I of the study. Feedback relating to usability and content, gathered using online think-aloud interviews and expert review groups, was used to refine the programmes prior to starting the trial. The results from phase I, which supports the robustness of the development of intervention content, will be published separately.

#### My Knee UK

This is a 12-week internet-based home exercise intervention that was modified from ‘My Knee Exercise’ (https://mykneeexercise.org.au), a 24-week intervention created and trialled in Australia by Nelligan *et al*.^15 26^ This intervention provides guidance, via a website, for participants to undertake an unsupervised, self-directed lower limb strengthening programme supported by online OA educational information and advice (table 2). The strengthening programme was reduced from the 24-week Australian version to what was considered a less burdensome 12-week programme by clinical members of the research team.^27^ The exercise programme was reviewed and refined by the research team and PPI members of the project advisory group, with input from an MSK physiotherapist expert review group.

**Table 2:** Summary of the My Knee UK rehabilitation programme

Webpage tab	Contents	
1. Home	Introductory video (from PCG) How to use the website and beginning your programme ‘Contact us’ for help tab	
2. My knee education	2.1. My knee education introduction 2.2. Understanding knee osteoarthritis (OA) 2.3. Understanding knee pain 2.4. Knee pain treatments 2.5. Exercise as treatment 2.6. Recommended exercise 2.7. Managing exercise pain 2.8. How to start in the exercise	
3. My knee strength	3.1. My knee strength introduction 3.2. How to start your knee exercises 3.3. Organise your exercise equipment 3.4. Tips for starting and sticking to exercise 3.5. Your mobile phone text message support **Exercise programme one (weeks 1–6)** Exercise instructions and videos (including self-tailoring exercise guides) (1) Sitting knee extension, (2) side steps, (3) calf raises **Exercise programme two (weeks 7–12)** Exercise instructions and videos (including self-tailoring exercise guides) (1) Sitting knee extension, (2) side steps, (3) calf raises, (4) mini (wall) squats, (5) chair rises (sit to stands)	
4. My knee activity	4.1. My knee activity introduction 4.2. Why increase physical activity? 4.3. How to increase physical activity 4.4. Track your daily steps 4.5. Activity pacing 4.6. Make a physical activity plan 4.7. Record your progress 4.8. Physical activity success stories (videos)	
5. My knee tools	Contains all the resources used throughout the website in one place	
**Examples of facilitator and barrier behaviour change messages** 29 30		
Facilitator	Hi (name), fitting in regular knee exercise is hard. The reason we are recommending weekly exercise is because exercise works best when it’s a regular thing. Exercising longer term can lead to lasting benefits in your knee health.	Do you have a goal you’d like to achieve if your knee improved? Think about what your goal is. Achieving your knee goals is the reward for doing the exercise programme.
Barrier	(Name) It’s okay to pull back the intensity of exercises if you’re feeling concerned. The important thing is that you do the exercises regularly. Gradually build up again as the knee becomes more stable and your confidence increases.	(Name) It can be hard to remember. We suggest making the exercises a habit. Set aside the same time each day to do them. It’s much harder to forget when something is a daily routine.

In this trial, three muscle strengthening exercises are introduced in programme one (weeks 1–6), and two additional strengthening exercises are added in programme two (weeks 7–12). The website (My Knee UK) encourages participants to perform their unsupervised home-based exercises at least three times a week and gives instructions about tailoring and progressing each exercise to their own ability/needs. Exercise logbooks and physical activity planners are available to download from the website to print or complete electronically. Participants are encouraged to access the website at leisure throughout the 12-week intervention and can phone/email a trial physiotherapist if they have any concerns or experience difficulties with the exercise programme.

Participants receive exercise-related behaviour change messages throughout the My Knee UK intervention via automated SMS delivered to their mobile phones (SMS Solutions Australia, Melbourne, Australia). The SMS library uses behaviour change theory (BCT) to identify and address key barriers to and facilitators of home-exercise programme adherence^28^ and has been shown to increase adherence to unsupervised home-based strengthening exercises.^29^ The 24-week SMS script, developed by Nelligan *et al*^30^ with input from academics, physiotherapists and consumers, was modified for use with the 12-week My Knee UK intervention by increasing the frequency of facilitator messages (from between 0 and 2 to between 1 and 3 per week), enabling the full BCT message library to be used (table 2).

#### Group E-Rehab

Group E-Rehab is a 12-week e-rehabilitation intervention comprising six internet-interactive education sessions and the same five lower limb strengthening exercises as My Knee UK. However, unlike those allocated to My Knee UK, Group E-Rehab participants receive seven group-based exercise sessions delivered remotely by a physiotherapist (table 3) via the videoconferencing platform Zoom (Zoom Video Communications, San Jose, USA) in weeks 1, 2, 3, 5, 7, 9 and 12 (table 3). The physiotherapist demonstrates/teaches the leg strengthening exercises (limited to three exercises in the first three classes), and conducts a 30 s chair sit-to-stand test^31^ remotely at the start of each class as a baseline measure and indication of progress. The physiotherapist monitors the sit-to-stand assessment alongside exercise quality, technique and effort during the classes, and uses these measures to tailor the exercises to meet each participant’s needs. Group sessions last 45–60 min, and each group contains four to seven participants.

**Table 3:** Summary of the Group E-Rehab rehabilitation programme

**Sway session 1: Pain and the knee joint** 1.1 About the knee joint Anatomy and physiology/using the knee joint 1.2 What is pain 1.3 What causes knee pain Knee osteoarthritis (OA)/some facts about OA 1.4 The pain cycle 1.5 Pain management Activity/conventional medicine Complementary and complementary medicine Other ways of managing knee pain 1.6 Test your knowledge (quiz)	**Sway session 2: Physical activity** 2.1 The benefits of exercise and physical activity Reducing the risk of falling How physically active am I? (quiz) 2.2 Building an active lifestyle Aerobic and cardiovascular exercise Physical activity recommendations 2.3 Exercise, general physical activity and joint pain Planning and recording physical activity Tracking your daily steps 2.4 The Group E-Rehab home exercise programme Format of the sessions/equipment Leg strengthening programme 2.5 Will I get new aches and pains if I exercise? 2.6 Physical activity quiz
**Sway Session 3: Goal setting** 3.1 Do my knee symptoms hold me back (quiz) 3.2 What are goals 3.3 Why and how should I set goals? 3.4 SMART goals Example of a SMART goal 3.5 Goal planning and implementation 3.6 Problem solving Problem solving example 3.7 Reward yourself 3.8 Goal setting and chronic pain (quiz)	**Sway session 4: Pacing skills** 4.1 Activity levels and pain 4.2 Flare ups 4.3 Balancing activity and rest Boom bust (overactivity/underactivity) cycle Activity rest cycle 4.4 Pacing your activity levels Putting pacing into practice 4.5 Pacing and chronic pain quiz (quiz) 4.6 Managing at work
**Sway Session 5: Communication and emotional well-being** 5.1 How well do I communicate with people? (quiz) 5.2 The importance of effective communication Making others aware (including professionals) 5.3 Communication styles Assertive communication 5.5 The importance of emotional well-being 5.6 Managing emotions, acceptance and feeling positive Getting and staying connected Distraction techniques/mindfulness 5.7 Managing setbacks 5.8 Emotions and chronic pain (quiz) 5.9 Online arthritis support	**Sway session 6: Staying healthy** 6.1 Good health 6.2 Healthy eating 6.3 Getting enough sleep Sleep and pain/sleeping well 6.4 Relax and unwind 6.5 Good health quiz (quiz) 6.6 Summary 6.7 What happens next? Continuing exercises and activities
**Home-based leg strengthening exercises** **Physiotherapist-led group-based exercise classes via Zoom in weeks 1, 2, 3, 5, 7, 9 and 12**. Weeks 1–6 (three exercises): (1) sitting knee extension, (2) side steps, (3) calf raises. Weeks 7–12 (five exercises): Exercises 1–3 plus (4) mini (wall) squats, (5) chair rises (sit to stands). 30 s sit-to-stand test done at the start of every physio-led Zoom class. Classes include time for discussing the self-directed Sway educational sessions.	

SMART, Specific, Measurable, Achievable, Relevant, and Time-Bound.

Interactive educational sessions developed for the Group E-Rehab intervention, available through the digital presentation programme Microsoft Sway,^32^ cover knee OA self-management and include optional quizzes with automated feedback, plus self-assessment questionnaires to make the sessions more personalised (table 3). Participants are advised that, although they can access the Sway education sessions in any order and at their leisure during the 12-week intervention, working through all six in the first 6 weeks will give them more opportunities to ask questions and/or discuss the contents during the online exercise classes. The physiotherapist provides dedicated time for this during each online class as well as reminding participants to engage with the education sessions and encouraging them to do their exercises at least three times each week. Participants are emailed exercise logbooks and physical activity planners to download.

Two Leeds Community MSK and Rehabilitation Service physiotherapists with current registration to practice in the UK were identified to deliver the Group E-Rehab intervention based on their clinical skills and experience. In preparation for the trial, both attended an external 1-day motivational interviewing training course (Et al Training, Leeds, UK). To facilitate consistent delivery of the intervention, particularly the exercise component, the physiotherapists completed practice Zoom classes and follow a comprehensive guidance document (written and provided by the study advanced practice physiotherapist (CC) and DG-W).

### Control arm

Participants allocated to the control group could receive any usual care intervention, ranging from no healthcare practitioner input to one or two physiotherapy sessions (delivered in-person or remotely by telephone or videoconferencing), to advice and guidance about self-management. The type of intervention that control group participants receive depends on their recruitment path.

### Enrolment

Participants are enrolled once they have been screened for eligibility by telephone (visit 1) and their informed, written postal consent has been countersigned by a delegated member of the research team (online supplemental 2). Eligible consenting participants complete a postal baseline questionnaire (visit 2) prior to randomisation.

10.1136/bmjopen-2022-063608.supp2 Supplementary data

### Randomisation and blinding

Participants are randomised (by DG-W) to one of the three groups using a 1:1:1 allocation ratio. Random allocation sequences with varying block length (3, 6 and 9), stratified by sex, are generated using an external password-protected web-based randomisation system (Sealed Envelope, London, UK). A dummy randomisation list,^33^ created using the same settings but different random seed number, was set up and then checked by the trial statistician (EMAH) before the trial randomisation list was created.

The nature of this feasibility trial means it is not possible to blind participants, physiotherapists delivering the Group E-Rehab intervention or the study team managing the trial. However, the trial statistician will remain blinded to group allocation until all preliminary data checks have been performed at a blinded data review meeting and the database has been locked.

### Trial data collection and outcomes

#### Follow-up visits

Follow-up postal questionnaires are sent out at 3 months (visit 3), which is the end of the intervention treatment period, and at 9 months (visit 4). Participants who withdraw from the trial early will not be replaced and will be requested to complete the next scheduled follow-up questionnaire. The schedule of enrolment and data collected during the trial is detailed in table 4.

**Table 4:** Standard Protocol Items: Recommendations for Interventional Trials schedule of enrolment, interventions and assessments.

	Study period			
	Enrolment	Randomisation	Follow-up	Close-out
Timepoint	−1	0	3 months ±4 weeks	9 months ±4 weeks
Enrolment				
Initial contact and study information	X			
Eligibility screen	X			
Informed consent	X			
Allocation		X		
Interventions				
Group E-Rehab			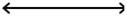	
‘My Knee UK’ Group			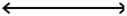	
Control group (duration of treatment will be variable)			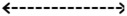	
Assessments				
Clinical information				
Age, gender, ethnicity		X		
Previous joint surgery		X		
Employment and physical activity		X		
Height and weight		X		
History of current knee pain		X		
General health and medication use		X	X	X
Patient-reported outcomes				
Joint pain manikin		X	X	X
Knee pain frequency (5-point scale)		X	X	X
Confidence and motivation to do exercises (11-point NRS)		X	X	X
Global change (7-point Likert scale)			X	X
WOMAC (Pain, function and stiffness)		X	X	X
ASES		X	X	X
HADS		X	X	X
Generic health status (EQ-5D-5L)		X	X	X
Health-related quality of life (SF-12)		X	X	X
Resource use			X	X

ASES, Arthritis Self-Efficacy Scale; EQ-5D-5L, European Quality of Life-Five Dimension-Five Level Scale; HADS, Hospital Anxiety and Depression Scale; NRS, numeric rating scale; SF-12, 12-Item Short Form Survey; WOMAC, The Western Ontario and McMaster Universities Osteoarthritis Index.

#### Outcomes

Patient-reported outcomes covering pain, function, health-related quality of life, coping and catastrophising, and confidence and motivation to do exercises will be measured alongside basic clinical findings at baseline and at the 3-month (12 weeks) and 9-month follow-up timepoints (figure 1 and table 4). These include validated outcome measures used with patients with OA and in knee OA clinical trials.^34–37^ Global change in overall pain and mobility/function and data on the number of contacts made with hospital and community health services, plus any costs incurred due to their knee pain (eg, prescription, travel costs to attend appointments), will be collected at the 3-month and 9-month timepoints. Primary and secondary outcomes have not been specified as this is a feasibility study.

#### Data management and monitoring

Identifiable data will be locked in a filing cabinet in the Leeds Institute of Rheumatic and Musculoskeletal Medicine (LIRMM) research offices or held in an encrypted file stored on a password-protected University of Leeds server, with access limited to the study team. Pseudonymised data will be entered onto a password-protected access database, developed in line with the LIRMM Data Quality Management System Standard Operating Procedures. Data will be periodically internally verified and audited. Data will be stored in a deidentified manner for 5 years after the final publication.

Physiotherapists will record the time taken to administer and lead the Group E-Rehab exercise classes and if a trial physiotherapist is contacted by a My Knee UK participant for help or advice, this will also be recorded. These data will contribute to estimating the cost of delivering the Group E-Rehab and My Knee UK interventions.

Descriptive data relating to exercise adherence relevant to each treatment arm will be collected at the end of the 12-week intervention phase. The web analytics service Google Analytics^38^ will be used to record the number of times each participant accesses the My Knee UK website and the duration of their website access. The weekly number of home-based exercise sessions completed (self-reported in response to the automated SMS message) will also be recorded. Group E-Rehab data will include the number of Zoom exercise classes attended and the number of Sway educational sessions accessed, along with the time spent engaged with each session.

#### Nested qualitative study

A subsample of consenting participants, purposively sampled by age, sex and having received the My Knee UK or Group E-Rehab intervention, will undergo individual in-depth remote (videoconference or telephone) interviews where the acceptability of the two e-rehabilitation interventions will be explored. Interviews will focus on participants’ experiences of being in the trial and will comprise questions specific to each intervention. It is anticipated around 20 individuals will be interviewed with the final number being determined once data saturation has been reached, which will be when there is consensus among the research team that minimal new information is being generated. Participants will be interviewed either on completion of the 3-month follow-up questionnaire (visit 3), or at the end of follow-up on completion of the 9-month questionnaire (visit 4).

Semistructured videoconference interviews with the two physiotherapists who delivered the Group E-Rehab Zoom classes will be conducted. This is to gain insight about the preparation they received, thoughts about its acceptability, their experience of delivering the intervention and any barriers to delivering it effectively that they identified.

### Planned analyses

The intention-to-treat participant population will be used. A full statistical analysis plan (SAP V.1.0, 04 January 2021) was written prior to commencement of recruitment.

#### Sample size

A total sample size of 90 participants (30 per arm), based on established principles for a feasibility study,^39^ will be adequate for evaluating feasibility and collecting sufficient data to inform the sample size in a definitive randomised controlled trial (RCT).

#### Feasibility and proof of concept

As a feasibility study, inferential statistics will be limited. Analysis will focus on descriptive statistics, including measures of frequency (eg, per cent), central tendency (eg, mean, median) and dispersion or variation (eg, SD, IQR, CI estimation), rather than formal hypothesis testing. Data will provide an estimate of recruitment and retention rates and the correlation between baseline and follow-up measurements, to inform the sample size required for a definitive RCT. In line with CONSORT^24^ the choice of primary outcome for the definitive study will be informed by the results of the feasibility study and guided by a previous study,^10^ but candidate variables will be The Western Ontario and McMaster Universities Osteoarthritis Index, pain and physical function domain scores. Published minimum clinically important differences (MCID) reported for the candidate primary outcomes will be used when calculating sample size.^40^ If the difference between the groups favours one or both intervention arms over the control arm, and the two-sided 85% CI around the difference includes the MCID, we will proceed to design a definitive trial provided feasibility criteria are met.^41^ Attrition will be examined to identify any factors that may be systematically affecting drop-out, and continuous measures of adherence within each treatment arm will be summarised. The cost of delivering the e-rehabilitation interventions will be estimated and alongside this, a descriptive report on the use of additional resources (eg, the type of resources, number of contacts and any costs incurred) will be produced.

#### Safety analyses

Adverse events (AEs) or serious adverse events (SAEs) will be recorded and coded to indicate the major event category but, as this is not a clinical trial of an investigational medicinal product, severity will not be graded. The frequency of all treatment-related AEs and SAEs recorded during the trial period will be displayed as the number of participants experiencing the AEs/SAEs, the percentage of participants and the number of AEs/SAEs will be presented both overall and by treatment arm.

#### Qualitative study

Data derived from the participant and semistructured physiotherapist interviews will be transcribed verbatim. Transcripts will be analysed using framework analysis.^42^

### Feasibility trial outcome

Data from the feasibility trial and follow-up nested qualitative study will be integrated to support the case for determining whether one or both e-rehabilitation interventions are feasible, acceptable and have the potential to be implemented in practice. The qualitative and quantitative data will enable the potential for My Knee UK and/or Group E-Rehab to be introduced as alternative models of service delivery to be explored. The feasibility trial will be deemed successful if the results demonstrate that (1) participants and physiotherapists find one or both intervention(s) acceptable (using data from the nested qualitative study), (2) it is possible to calculate a manageable sample size for use in a definitive RCT, (3) attrition at visit 3 (3 months) is no more than 30% and (4) at least 40% of eligible patients are recruited to the trial. If one or both e-rehabilitation interventions are acceptable, the intention is to develop the protocol for and conduct a definitive RCT with a health economic component. This will be sufficiently powered for testing the wider use of My Knee UK and/or Group E-Rehab (depending on feasibility trial outcome) as a prescribed treatment for individuals with persistent knee pain.

## Discussion

The growing prevalence of persistent knee pain and OA requires the development and implementation of effective and accessible treatments that enable these patients to manage their symptoms. These treatments should be convenient for patients and should not overburden local MSK physiotherapy services. Current evidence suggests that for chronic conditions, such as OA, digital technologies can be used to deliver self-management programmes electronically.^9 43^

This trial aims to evaluate the feasibility and acceptability of two different e-rehabilitation interventions (Group E-Rehab and My Knee UK) in individuals with persistent knee pain. Education, advice about increasing general physical activity levels and strengthening exercises that can be tailored to individual needs are pivotal components of both interventions. Muscle strengthening programmes are beneficial in reducing pain and improving physical function in people with knee OA.^44^ In this trial, the home-based lower-limb strengthening exercise programme is either prescribed and monitored by a physiotherapist as a group-based intervention using videoconferencing (Group E-Rehab) or it is self-directed (accessed via the My Knee UK website). It is believed that the e-rehabilitation interventions under investigation in this trial will provide patients with access to the digital tools and resources needed for self-managing their knee pain and symptoms, and that one or both interventions could eventually be implemented within the NHS.

### Ethics and dissemination

This feasibility trial and current protocol (v.5.0, 25 January 2022) was approved by the West of Scotland Research Ethics Committee 5 (REC5), reference number 20/WS/0006 prior to commencing recruitment, which is ongoing. The study is sponsored by the University of Leeds (Research Integrity and Governance), UK and is registered with the ISRCTN (online supplemental 3). All individuals assisting with the trial will be informed of any protocol amendments, which will be approved by the Sponsor before being submitted to the REC/HRA for approval. The results will be disseminated to the study grant funder, submitted for publication in peer-reviewed journals and where requested, a summary of the study findings will be disseminated to study participants. No study participants will be identifiable in the study results.

10.1136/bmjopen-2022-063608.supp3 Supplementary data

## Supplementary Material

Reviewer comments

Author's manuscript
