# Supplementary material for: Evaluation of two electronic-rehabilitation programmes for persistent knee pain: protocol for a randomised feasibility trial
Source: BMJ Open. 2022 Jun 3;12(6):e063608. doi: 10.1136/bmjopen-2022-063608 (PMC9171213; doi:10.1136/bmjopen-2022-063608)
Supplement: Supplementary data [file bmjopen-2022-063608supp001.pdf]

SUPPLEMENT 1

Protocol modifications made to mitigate the effects of the Covid-19 Pandemic

1. Changes arising from the Covid-19 pandemic mean that routine physiotherapy appointments are now more likely to be remote rather than face-to-face. Currently, most appointments are taking place over the telephone or in some case, via video consultation, rather than being face-to-face in a clinic. Prior to starting the recruitment process for the feasibility trial, the protocol and participant documents were amended to reflect these changes. This means that potential participants will be fully aware that if they enter the study and are allocated to the control group, if they are current patients who will receive usual care, then their physiotherapy appointments are unlikely to be face-to-face, particularly if Covid-19 restrictions are still in place.

2. Given the current and potential future restrictions arising from the Covid-19 pandemic, interviews with the physiotherapists and sub-sample of participants from the trial during the nested qualitative study phase will be done remotely.

3. Current Covid-19 restrictions mean that only skeleton staff members from the research team were physically going into the office. This would have caused a delay in responding to the initial postal response to enquiries from potential participants. To avoid this, the invitation letter was amended slightly so that if anyone receiving the study information is interested in joining the study, they are asked to contact the study team via email or phone instead of returning a paper contact form.

4. A delayed start due to Covid-19 has resulted in shortened timeframe for recruitment, with the risk that reaching the target of 90 participants within the study timeframe would not be achievable. To mitigate this, additional ways of identifying and recruiting participants were added to the protocol. This included:

Groves-Williams D, *et al. BMJ Open* 2022; 12:e063608. doi: 10.1136/bmjopen-2022-063608

- Searching local Trust MSK service records for potentially eligible patients who have previously received physio for knee pain.
- Using Participant identification Centres (PICs) who have the appropriate approval and model agreement in place to carrying out a search of their patient records database to identify individuals that meet a study's eligibility criteria.
- A database search of participants from previous University of Leeds studies who have consented to be contacted about future relevant research studies.
- A media campaign with physical posters/flyers in clinics, waiting rooms or local community centres, and an electronic poster/advert that can be distributed digitally (e.g. email, University or Trust websites), and used via social media.

Participants recruited via MSK waiting lists or clinics (current patients) will continue to receive a physio appointment (usual care) if they are randomised to the control group or if they subsequently withdraw. However, the amendment will affect participants identified via service records, database searches, media campaigns or PICs if they are randomised to the control group or later withdraw from the trial. Participants recruited using these methods (i.e. not via the MSK waiting list or clinics), will be asked to continue with whatever exercises, treatment, or self-management they were doing before joining the trial.

Groves-Williams D, *et al. BMJ Open* 2022; 12:e063608. doi: 10.1136/bmjopen-2022-063608
