# Supplementary material for: Evaluation of two electronic-rehabilitation programmes for persistent knee pain: protocol for a randomised feasibility trial
Source: BMJ Open. 2022 Jun 3;12(6):e063608. doi: 10.1136/bmjopen-2022-063608 (PMC9171213; doi:10.1136/bmjopen-2022-063608)
Supplement: Supplementary data [file bmjopen-2022-063608supp002.pdf]

SUPPLEMENT 2

Sample participant consent form for the Phase 2 trial

UNIVERSITY OF LEEDS

Leeds Institute of Rheumatic and Musculoskeletal Medicine
2nd Floor, Chapel Allerton Hospital
Chapeltown Road, Leeds, LS7 4SA
Email: e.rehab@leeds.ac.uk
Tel: 0113 392 4965

Participant Identification Number [ ]

CONSENT FORM (IRAS Reference: 269827)

Evaluation of electronic-rehabilitation programmes for chronic knee pain

Phase 2: Feasibility trial

Please initial each box

1	I confirm that I have read the information sheet dated 18/05/2021 (version 3.0) for the above study. I have had the opportunity to consider the information, ask questions and have had these answered satisfactorily.	
2	I understand that my participation is voluntary and that I am free to withdraw at any time without giving any reason, and without my legal rights or medical care being affected.	
3	I understand that, even if I withdraw from this study, the information already collected from me will be used in analysing the results of this study.	
4	I understand that the group sessions will be recorded and any identifiable audio and visual data from these recordings will be stored securely and not shared.	
5	I understand that the information collected during this study may be used to support other research in the future and may be shared anonymously with other researchers.	
6	I agree to my personal information being stored for the purposes of this study. I understand that any information which could identify me will be kept strictly confidential.	
7	I understand that relevant sections of the data collected during the study, may be looked at by individuals from the University of Leeds (sponsors) or from regulatory authorities where it is relevant to my taking part in this research.	
8	I understand that my GP will be informed about my participation in this study.	
9	I agree to take part in the above study.	

After initialling the boxes, please print and sign your name (participant), add the date, and fill out the participant contact details on the next page before returning the form.

Name of Participant (PRINTED)	Date	Signature

Please remember to fill in your contact details at the bottom of the page.

The contact slip will be removed and destroyed after we have put your details on to our secure Subject Screening Log, which is used by the research team for contacting you about the study.

Name of Researcher	*Date	Signature

When completed: 1 for participant; 1 for researcher site file. *Will be signed by researcher on a different day.

✂...

Participant Contact Details

Telephone Number(s): ____

Email Address: ____

IRAS ID	*Project Title*	*Document Type*	*Version #*	*Date*	*Page*
269827	E-rehab for Knee Pain	Phase 2 Trial Patient Consent Form	4.0	10/09/2021	**Page 2 of 2**

Groves-Williams D, *et al. BMJ Open* 2022; 12:e063608. doi: 10.1136/bmjopen-2022-063608
