# Supplementary material for: Evaluation of two electronic-rehabilitation programmes for persistent knee pain: protocol for a randomised feasibility trial
Source: BMJ Open. 2022 Jun 3;12(6):e063608. doi: 10.1136/bmjopen-2022-063608 (PMC9171213; doi:10.1136/bmjopen-2022-063608)
Supplement: Supplementary data [file bmjopen-2022-063608supp003.pdf]

SUPPLEMENT 3

Trial registration

Data category	Information
Registry identifying number	ISRCTN15564385
Date of registration	07/02/2020
Prospective/Retrospective	Prospectively registered
Additional identifiers	CPMS 43473, IRAS 269827
Sources of monetary support	Versus Arthritis; National Institute for Health Research (NIHR) (UK)
Sponsor	University of Leeds
Contact information	Dr Dawn Groves-Williams; d.groves-williams@leeds.ac.uk
	Dr Sarah Kingsbury; s.r.kingsbury@leeds.ac.uk
Short/public title	E-rehab for Knee Pain
Scientific title	Evaluation of electronic-rehabilitation programmes for chronic knee pain
Countries of recruitment	United Kingdom
Condition category	Musculoskeletal Diseases
Condition	Chronic knee pain
Interventions	Access to **My Knee UK** website containing exercise videos and self-management resources for 12 weeks. Support via SMS messages
	**Group E-Rehab**: access to online educational and self-management resources plus 7 online group physiotherapy classes over 12-weeks
	**Control Group**: depending on their recruitment path – will receive usual physiotherapy care or continue with usual self-management
Key inclusion and exclusion criteria	Type: Adults ≥ 45 years; both sexes; no healthy volunteers
	Inclusion: knee pain > 3 months and on most days of previous month; activity-related joint pain; pain during walking ≥4 on an 11-point scale
	Exclusion: Inflammatory arthritis/gout; Joint replacement in study knee; injection within last month; arthroscopy within last 3-months
Study design	Mixed methods interventional randomised controlled feasibility trial with follow-up interviews
	Primary: Interventional; Secondary: Randomised controlled trial; Trial setting: Community; Trial type: treatment
Target sample size	90 (30 in each group)
Date of first enrolment	22/04/2021
Recruitment status	Recruiting
Overall trial status	Ongoing
Key outcomes<br>(Primary and secondary outcomes have not been specified due to this being a feasibility study).	Participants and physiotherapists find e-rehabilitation feasible and acceptable, and it can be administered successfully; calculation of a sample size that can be achieved in a main trial; max. of 30% attrition at 3-months; ≥ 40-50% of eligible participants are recruited.<br>Patient-reported outcome domains are: pain; quality of life; pain coping and catastrophising; resource use; confidence and motivation to do exercises; global change in overall pain and mobility/function.

Groves-Williams D, *et al. BMJ Open* 2022; 12:e063608. doi: 10.1136/bmjopen-2022-063608
