# Supplementary material for: Evaluation of two electronic-rehabilitation programmes for persistent knee pain: protocol for a randomised feasibility trial
Source: BMJ Open. 2022 Jun 3;12(6):e063608. doi: 10.1136/bmjopen-2022-063608 (PMC9171213; doi:10.1136/bmjopen-2022-063608)
Supplement: Author's manuscript [file bmjopen-2022-063608.draft_revisions.pdf]

BMJ Open is committed to open peer review. As part of this commitment we make the peer review history of every article we publish publicly available.

When an article is published we post the peer reviewers' comments and the authors' responses online. We also post the versions of the paper that were used during peer review. These are the versions that the peer review comments apply to.

The versions of the paper that follow are the versions that were submitted during the peer review process. They are not the versions of record or the final published versions. They should not be cited or distributed as the published version of this manuscript.

BMJ Open is an open access journal and the full, final, typeset and author-corrected version of record of the manuscript is available on our site with no access controls, subscription charges or pay-per-view fees (http://bmjopen.bmj.com).

If you have any questions on BMJ Open's open peer review process please email info.bmjopen@bmj.com

BMJ Open

Evaluation of two electronic-rehabilitation programmes for persistent knee pain: protocol for a randomised feasibility trial

Journal:	*BMJ Open*
Manuscript ID	bmjopen-2022-063608
Article Type:	Protocol
Date Submitted by the Author:	04-Apr-2022
Complete List of Authors:	Groves-Williams, Dawn; University of Leeds Faculty of Medicine and Health, Leeds Institute of Rheumatic and Musculoskeletal Medicine, Chapel Allerton Hospital<br>McHugh, Gretl; University of Leeds School of Healthcare<br>Bennell, Kim; The University of Melbourne Centre for Health Exercise and Sports Medicine, Department of Physiotherapy<br>Comer, Christine; Leeds Community Healthcare NHS Trust, Musculoskeletal and Rehabilitation Service<br>Hensor, Liz; University of Leeds Faculty of Medicine and Health, Leeds Institute of Rheumatic and Musculoskeletal Medicine, Chapel Allerton Hospital; NIHR Leeds Biomedical Research Centre<br>Conner, Mark; University of Leeds School of Psychology<br>Nelligan, Rachel; The University of Melbourne Centre for Health Exercise and Sports Medicine, Department of Physiotherapy<br>Hinman, Rana; University of Melbourne,<br>Kingsbury, Sarah; University of Leeds Faculty of Medicine and Health, Leeds Institute of Rheumatic and Musculoskeletal Medicine, Chapel Allerton Hospital; NIHR Leeds Biomedical Research Centre,<br>Conaghan, Philip; University of Leeds Faculty of Medicine and Health, Leeds Institute of Rheumatic and Musculoskeletal Medicine, Chapel Allerton Hospital; NIHR Leeds Biomedical Research Centre
Keywords:	PAIN MANAGEMENT, Rheumatology < INTERNAL MEDICINE, World Wide Web technology < BIOTECHNOLOGY & BIOINFORMATICS, Telemedicine < BIOTECHNOLOGY & BIOINFORMATICS

SCHOLARONE™
Manuscripts

Evaluation of two electronic-rehabilitation programmes for persistent knee pain: protocol for a randomised feasibility trial

Dawn Groves-Williams [a], Gretl A. McHugh [c], Kim L. Bennell [d], Christine Comer [e,a], Elizabeth M A Hensor[a,b], Mark Conner [f], Rachel K. Nelligan [d], Rana S Hinman [d], Sarah R. Kingsbury [a,b,*], Philip G. Conaghan [a,b,*]

Corresponding author.

Assoc Prof Sarah Kingsbury, Leeds Institute of Rheumatic and Musculoskeletal Medicine (LIRMM), 2nd Floor, Chapel Allerton Hospital, Chapeltown Road, Leeds LS7 4SA, UK.

Email: s.r.kingsbury@leeds.ac.uk; Telephone: +44 113 392 4883

Author affiliations

[a] Leeds Institute of Rheumatic and Musculoskeletal Medicine (LIRMM), School of Medicine, Chapel Allerton Hospital, Leeds.

[b] NIHR Leeds Biomedical Research Centre (BRC), Chapel Allerton Hospital, Leeds, UK.

[c] School of Healthcare, University of Leeds, Leeds, UK.

[d] Centre for Health Exercise and Sports Medicine, Department of Physiotherapy, School of Health Sciences, The University of Melbourne, Melbourne, Australia.

[e] Leeds Community Healthcare NHS Trust, Wetherby Health Centre, Leeds, UK.

[f] School of Psychology, University of Leeds, Leeds, UK.

[*] These authors contributed equally.

KEYWORDS

Pain management

Rheumatology

For peer review only - http://bmjopen.bmj.com/site/about/guidelines.xhtml

World wide web technology

Telemedicine

WORD COUNT

Abstract: 298

Manuscript: 3782 (excl. title page, abstract, table contents, references)

For peer review only

For peer review only - http://bmjopen.bmj.com/site/about/guidelines.xhtml

ABSTRACT

Introduction

Persistent, knee pain is a common cause of disability. Education and exercise treatment are advocated in all clinical guidelines; however, the increasing prevalence of persistent knee pain presents challenges for health services regarding appropriate and scalable delivery of these treatments. Digital technologies may help address this, and this trial will evaluate the feasibility and acceptability of two electronic-rehabilitation (e-rehabilitation) interventions: 'My Knee UK' and 'Group E-Rehab'.

Methods and analysis

This protocol describes a non-blinded, randomised feasibility trial with three parallel groups. The trial aims to recruit 90 participants (45 years or older) with a history of persistent knee pain consistent with a clinical diagnosis of knee osteoarthritis (OA). Participants will be randomly assigned in a 1:1:1 allocation ratio. The 'My Knee UK' intervention arm will receive a self-directed unsupervised Internet-based home exercise programme plus short message service (SMS) support (targeting exercise behaviour change) for 12-weeks; the 'Group E-Rehab' intervention arm will receive group-based physiotherapist-prescribed home exercises delivered via videoconferencing accompanied by Internet-interactive educational sessions for 12-weeks; the control arm will receive usual physiotherapy care or continue with their usual self-management (depending on their recruitment path). Feasibility variables, patient-reported outcomes and clinical findings measured at baseline, three and nine-months will be assessed and integrated with qualitative interview data from a sub-set of Group E-Rehab and My Knee UK participants. If considered feasible and acceptable, a definitive randomised controlled trial can be conducted to investigate the clinical- and cost-effectiveness of one or both interventions with a view to implementation in routine care.

For peer review only

For peer review only - http://bmjopen.bmj.com/site/about/guidelines.xhtml

Ethics and dissemination

The trial was approved by the West of Scotland Research Ethics Committee 5 (Reference: 20/WS/0006). The results of the study will be disseminated to study participants, the study grant funder, and will be submitted for publication in peer-reviewed journals.

Trial registration number

ISRCTN: 15564385.

For peer review only

ARTICLE SUMMARY

Strengths and limitations of this study

- The interventions being investigated are accessed/delivered remotely and do not require any face-to-face contact. This also means that participants do not have to travel to appointments, which can be beneficial for some individuals.
- Patient and public involvement alongside expert review conducted during the first phase ensured the content, appearance, and delivery of the interventions during the trial phase were tailored to the needs of individuals with persistent knee pain.
- The mixed methods approach to data collection and analysis will provide a greater understanding of the feasibility and acceptability of the two digital health interventions.
- It is not possible to blind the participants, physiotherapists or the trials staff administering the study, however, the trial statistician will remain blinded to group allocation until the database has been locked.
- Participants need to have an active email account , a mobile phone, and access to a computer or tablet with Internet access suitable for receiving/making video calls, which may exclude some individuals.

For peer review only

INTRODUCTION

Persistent or chronic knee pain, often with associated stiffness and functional limitations, is a common problem in older and middle-aged adults.[1,2] A leading cause is osteoarthritis (OA) and in 2020, the pooled global prevalence of knee OA in individuals aged 40 and over was reported to be around 23%, with a positive correlation between prevalence and increased age.[3] Approximately 10% of adults in the United Kingdom (UK) have a clinical diagnosis of OA, with symptomatic knee OA being the commonest site.[4] It is estimated that by 2035 the number of people with knee OA in the UK could reach 8.3 million.[5] Exercise and access to appropriate education are advocated in all clinical guidelines as core treatments for persistent knee pain. However, with the increasing prevalence of persistent knee pain and current treatment delivery strategies not always addressing patient needs, managing these patients is challenging.[6]

For peer review only

Physiotherapists are key in providing education, exercises, and self-management support to improve symptoms and function for individuals with knee OA.[7] It has previously been observed that physiotherapists can effectively deliver OA rehabilitation interventions remotely using video technologies,[8–10] which is increasingly important given the adoption of telehealth during the Covid-19 pandemic[11–13]. It has been reported that since the onset of Covid-19, 88% of 50-64 year olds, 75% of 65-74 year olds and 46% of those aged 75 or over use the Internet almost every day or every day.[14] These data support the assumption that some, but not all older individuals could access Internet-based electronic interventions.

Digital health interventions for knee pain have been developed and delivered by physiotherapists in Australia; and there is evidence to suggest they can improve knee pain and function[9,15] and are generally accepted by patients with knee OA.[16] Such digital health interventions may be useful in other large health services (such as in the UK) as an alternative to “in person” clinic appointments, particularly where physiotherapy services are overstretched[17] and waiting times are prolonged

For peer review only - http://bmjopen.bmj.com/site/about/guidelines.xhtml

due to the Covid-19 pandemic,[18] or where individuals are unable to attend physiotherapy sessions due to mobility problems and/or lack of transport.[19–21]

Aims and objectives

This trial aims to evaluate the feasibility and acceptability of two different electronic-rehabilitation (e-rehabilitation) interventions in individuals with persistent knee pain. One comprises a self-directed Internet-based home exercise programme with exercise behaviour change support provided via automated SMS ('My Knee UK'). The second is a group-based home exercise programme with Internet-interactive education sessions ('Group E-Rehab'), where the home exercise programme is prescribed and monitored by a physiotherapist using videoconferencing. The trial will also explore the effect of each e-rehabilitation programme on pain and other symptoms compared to the control arm and provide a report on any additional resources (e.g. additional NHS/private healthcare services) used by participants during the trial.

METHODS

Trial design

This is an unblinded, single-centre, randomised feasibility trial with three parallel arms (two e-rehabilitation treatment arms and one control arm). The study protocol was designed to conform to the Standard Protocol Items: Recommendations for Interventional Trials (SPIRIT) guidelines[22,23] and the extension of the Consolidated Standards of Reporting Trials (CONSORT) statement for randomised pilot and feasibility trials.[24] Trial phases are outlined in Figure 1.

*(Insert **Figure 1:** Study flow diagram.)*

For peer review only - http://bmjopen.bmj.com/site/about/guidelines.xhtml

In line with the recently published CONSERVE 2021 Statement and guidelines,[25] several protocol changes were made in response to the Covid-19 pandemic (Supplement 1).

Patient and Public Involvement

Members of the NIHR Leeds Biomedical Research Centre Patient and Public Involvement (PPI) group provided input into the trial design and outcome measures through focus group discussions during the funding application stages. PPI was subsequently used to provide input into the refinement of the two e-rehabilitation programmes during the first phase of the study. Additionally, two PPI representatives are members of the Project Advisory Group and help review content and assist with key decisions throughout the trial.

Participants

The target population is adults with persistent knee pain who meet the eligibility criteria (Table 1). Potential participants have either been referred to musculoskeletal (MSK) services for assessment or have previously received physiotherapy for persistent knee pain; are participants from past studies that have consented to future contact; have responded to a media campaign advertising for volunteers (Figure 1).

Table 1: Trial eligibility criteria.

Inclusion Criteria	Exclusion Criteria
Adults > 45 years	Inflammatory arthritis (including gout)
Knee pain > 3 months and on most days of previous month	A joint replacement in the study knee
Knee pain during walking ≥4 on an 11-point numerical rating scale[26]	An injection into the study knee joint within the last month
Activity-related knee joint pain	An arthroscopy of the study knee joint in the last three months
Has a mobile phone, active email account, and computer with Internet access suitable for receiving and making video calls if required.	Enrolled in another research study involving an intervention for OA treatment or management
	Unable to comply with the study protocol
	Unable to understand written and spoken English.

For peer review only

For peer review only - http://bmjopen.bmj.com/site/about/guidelines.xhtml

Digital health intervention treatment arms

The e-rehabilitation interventions were adapted for use in the UK (My Knee UK) or developed (Group E-Rehab) during Phase 1 of the study. Feedback relating to usability and content, gathered using online think-aloud interviews and expert review groups, was used to refine the programmes prior to starting the trial. The results from Phase 1, which supports the robustness of the development of intervention content, will be published separately.

My Knee UK

This is a 12-week Internet-based home exercise intervention that was modified from 'My Knee Exercise' (https://mykneeexercise.org.au), a 24-week intervention created and trialled in Australia by Nelligan et al.[15,27] This intervention provides guidance, via a website, for participants to undertake an unsupervised, self-directed lower limb strengthening programme supported by online OA educational information and advice (Table 2). The strengthening programme was reduced from the 24-week Australian version to what was considered a less burdensome 12-week programme by clinical members of the research team.[28] The exercise programme was reviewed and refined by research team and PPI members of the Project Advisory group, with input from an MSK physiotherapist expert review group.

In this trial, three muscle strengthening exercises are introduced in Programme One (weeks 1-6), and two additional strengthening exercises are added in Programme Two (weeks 7-12). The website (My Knee UK) encourages participants to perform their unsupervised home-based exercises at least three times a week and gives instructions about tailoring and progressing each exercise to their own ability/needs. Exercise logbooks and physical activity planners are available to download from the website to print or complete electronically. Participants are encouraged to access the website at leisure throughout the 12-week intervention and can phone/email a trial physiotherapist if they have any concerns or experience difficulties with the exercise programme.

For peer review only - http://bmjopen.bmj.com/site/about/guidelines.xhtml

Participants receive exercise-related behaviour change messages throughout the My Knee UK intervention via automated SMS delivered to their mobile phones (SMS Solutions Australia, Melbourne, Australia). The SMS library uses behaviour change theory to identify and address key barriers to and facilitators of home-exercise programme adherence[29] and has been shown to increase adherence to unsupervised home-based strengthening exercises.[30] The 24-week SMS script, developed by Nelligan et al.[31] with input from academics, physiotherapists, and consumers, was modified for use with the 12-week My Knee UK intervention by increasing the frequency of facilitator messages (from between 0 & 2 to between 1 & 3 per week), enabling the full BCT message library to be used (Table 2).

For peer review only

For peer review only - http://bmjopen.bmj.com/site/about/guidelines.xhtml

Table 2: Summary of the My Knee UK rehabilitation programme.

Webpage Tab	Contents
**1. Home**	Introductory video (from Prof Conaghan)<br>How to use the website and beginning your programme<br>'Contact Us' for help tab
**2. My Knee Education**	2.1. My knee education introduction<br>2.2. Understanding knee osteoarthritis (OA)<br>2.3. Understanding knee pain<br>2.4. Knee pain treatments<br>2.5. Exercise as treatment<br>2.6. Recommended exercise<br>2.7. Managing exercise pain<br>2.8. How to start in the exercise
**3. My Knee Strength**	3.1. My knee strength introduction<br>3.2. How to start your knee exercises<br>3.3. Organise your exercise equipment<br>3.4. Tips for starting and sticking to exercise<br>3.5. Your mobile phone text message support<br>**Exercise Programme One (weeks 1-6)**<br>Exercise instructions and videos (incl. self-tailoring exercise guides)<br>a) Sitting knee extension, b) Side steps, c) Calf raises.<br>**Exercise Programme One (weeks 2-7)**<br>Exercise instructions and videos (incl. self-tailoring exercise guides)<br>a) Sitting knee extension, b) Side steps, c) Calf raises,<br>d) Mini (wall) squats, e) Chair rises (sit-to-stands).
**4. My Knee Activity**	4.1. My knee activity introduction<br>4.2. Why increase physical activity?<br>4.3. How to increase physical activity<br>4.4. Track your daily steps<br>4.5. Activity pacing<br>4.6. Make a physical activity plan<br>4.7. Record your progress<br>4.8. Physical activity success stories (videos)
**5. My Knee Tools**	Contains all the resources used throughout the website in one place.

**Examples of facilitator and barrier behaviour change messages**[30,31]		
**Facilitator**	Hi [name], fitting in regular knee exercise is hard. The reason we are recommending weekly exercise is because exercise works best when it's a regular thing. Exercising longer term can lead to lasting benefits in your knee health.	Do you have a goal you'd like to achieve if your knee improved? Think about what your goal is. Achieving your knee goals is the reward for doing the exercise programme.
**Barrier**	[Name] It's okay to pull back the intensity of exercises if you're feeling concerned. The important thing is that you do the exercises regularly. Gradually build up again as the knee becomes more stable & your confidence increases.	[Name] It can be hard to remember. We suggest making the exercises a habit. Set aside the same time each day to do them. It's much harder to forget when something is a daily routine.

For peer review only

For peer review only - http://bmjopen.bmj.com/site/about/guidelines.xhtml

Group E-Rehab

Group E-Rehab is a 12-week e-rehabilitation intervention comprising six Internet-interactive education sessions and the same five lower limb strengthening exercises as My Knee UK. However, unlike those allocated to My Knee UK, Group E-Rehab participants receive seven group-based exercise sessions delivered remotely by a physiotherapist (Table 3) via the videoconferencing platform Zoom (Zoom Video Communications Inc., San Jose, USA) in weeks 1, 2, 3, 5, 7, 9, and 12 (Table 3). The physiotherapist demonstrates/teaches the leg strengthening exercises (limited to three exercises in the first three classes), and conducts a 30-second chair sit-to-stand test[32] remotely at the start of each class as a baseline measure and indication of progress. The physiotherapist monitors the sit-to-stand assessment alongside exercise quality, technique, and effort during the classes, and uses these measures to tailor the exercises to meet each participant's needs. Group sessions last 45-60 minutes, and each group contains four-seven participants.

Interactive educational sessions developed for the Group E-Rehab intervention, available through the digital presentation programme Microsoft Sway,[33] cover knee OA self-management and include optional quizzes with automated feedback, plus self-assessment questionnaires to make the sessions more personalised (Table 3). Participants are advised that, although they can access the Sway education sessions in any order and at their leisure during the 12-week intervention, working through all six in the first 6-weeks will give them more opportunities to ask questions and/or discuss the contents during the online exercise classes. The physiotherapist provides dedicated time for this during each online class as well as reminding participants to engage with the education sessions and encouraging them to do their exercises at least three times each week. Participants are emailed exercise logbooks and physical activity planners to download.

Two Leeds Community MSK and Rehabilitation Service physiotherapists with current registration to practice in the UK were identified to deliver the Group E-Rehab intervention based on their clinical skills and experience. In preparation for the trial, both attended an external one-day motivational

For peer review only - http://bmjopen.bmj.com/site/about/guidelines.xhtml

interviewing training course (Et al Training, Leeds, UK). To facilitate consistent delivery of the intervention, particularly the exercise component, the physiotherapists completed practice Zoom classes and follow a comprehensive guidance document (written and provided by the study Advanced Practice Physiotherapist [CC] and DGW).

For peer review only

For peer review only - http://bmjopen.bmj.com/site/about/guidelines.xhtml

Table 3: Summary of the Group E-Rehab rehabilitation programme.

**Sway Session 1: Pain and the Knee Joint**<br>1.1 About the Knee joint<br>Anatomy and physiology / Using the knee joint<br>1.2 What is Pain<br>1.3 What causes knee pain<br>Knee Osteoarthritis (OA) / Some facts about OA<br>1.4 The Pain cycle<br>1.5 Pain Management<br>Activity / Conventional medicine<br>Complementary and alternative medicine<br>Other ways of managing knee pain<br>1.6 Test your knowledge (quiz)	**Sway Session 2: Physical Activity**<br>2.1 The Benefits of Exercise and Physical Activity<br>Reducing the risk of falling<br>How physically active am I? (quiz)<br>2.2 Building an active lifestyle<br>Aerobic and cardiovascular exercise<br>Physical Activity Recommendations<br>2.3 Exercise, general physical activity, and Joint Pain<br>Planning and recording physical activity<br>Tracking your daily steps<br>2.4 The Group E-Rehab Home Exercise Programme<br>Format of the sessions / equipment<br>Leg strengthening programme<br>2.5 Will I get New Aches and Pains if I exercise?<br>2.6 Physical activity quiz
**Sway Session 3: Goal Setting**<br>3.1 Do my knee symptoms hold me back (quiz)<br>3.2 What are goals<br>3.3 Why and How should I set goals?<br>3.4 SMART goals<br>Example of a SMART goal<br>3.5 Goal Planning and Implementation<br>3.6 Problem Solving<br>Problem solving example<br>3.7 Reward yourself<br>3.8 Goal setting and chronic pain (quiz)	**Sway Session 4: Pacing Skills**<br>4.1 Activity levels and Pain<br>4.2 Flare ups<br>4.3 Balancing Activity and Rest<br>Boom Bust (Overactivity/underactivity) cycle<br>Activity rest cycle<br>4.4 Pacing your activity levels<br>Putting Pacing into practice<br>4.5 Pacing and chronic pain quiz (quiz)<br>4.6 Managing at Work
**Sway Session 5: Communication and Emotional Wellbeing**<br>5.1 How well do I communicate with people? (quiz)<br>5.2 The importance of Effective Communication<br>Making others aware (inc. professionals)<br>5.3 Communication Styles<br>Assertive Communication<br>5.5 The Importance of Emotional Wellbeing<br>5.6 Managing Emotions, Acceptance, and Feeling Positive<br>Getting and Staying Connected<br>Distraction techniques / Mindfulness<br>5.7 Managing Setbacks<br>5.8 Emotions and Chronic Pain (quiz)<br>5.9 Online Arthritis Support	**Sway Session 6: Staying Healthy**<br>6.1 Good Health<br>6.2 Healthy Eating<br>6.3 Getting Enough Sleep<br>Sleep and Pain / Sleeping well<br>6.4 Relax and Unwind<br>6.5 Good Health Quiz (quiz)<br>6.6 Summary<br>6.7 What Happens Next?<br>Continuing exercises and activities
**Home-based Leg Strengthening Exercises**<br>**Physiotherapist-led group-based exercise classes via Zoom in weeks 1, 2, 3, 5, 7, 9, and 12.**<br>Weeks 1-6 (3 exercises): a) Sitting knee extension, b) Side steps, c) Calf raises (weeks 1-6).<br>Weeks 7-12 (5 exercises): Exercises a to c plus - d) Mini (wall) squats, e) Chair rises (sit-to-stands).<br>• 30 second sit-to-stand test done at the start of every physio-led Zoom class.<br>• Classes include time for discussing the self-directed Sway educational sessions.	

For peer review only

For peer review only - http://bmjopen.bmj.com/site/about/guidelines.xhtml

Control arm

Participants allocated to the control group could receive any usual care intervention, ranging from no healthcare practitioner input to one or two physiotherapy sessions (delivered in-person or remotely by telephone or videoconferencing), to advice and guidance about self-management. The type of intervention that control group participants receive depends on their recruitment path.

Enrolment

Participants are enrolled once they have been screened for eligibility by telephone (visit 1) and their informed, written postal consent has been countersigned by a delegated member of the research team (Supplement 2). Eligible consenting participants complete a postal baseline questionnaire (visit 2) prior to randomisation.

Randomisation and blinding

Participants are randomised (by DGW) to one of the three groups using a 1:1:1 allocation ratio. Random allocation sequences with varying block length (3, 6 and 9), stratified by sex, are generated using an external password-protected web-based randomisation system (Sealed Envelope Ltd. London, UK). A dummy randomisation list[34], created using the same settings but different random seed number, was set up and then checked by the trial statistician (EMAH) before the trial randomisation list was created.

The nature of this feasibility trial means it is not possible to blind participants, physiotherapists delivering the Group E-Rehab intervention, or the study team managing the trial. However, the trial statistician will remain blinded to group allocation until all preliminary data checks have been performed at a blinded data review meeting and the database has been locked.

For peer review only

For peer review only - http://bmjopen.bmj.com/site/about/guidelines.xhtml

Trial data collection and outcomes

Follow-up visits

Follow-up postal questionnaires are sent out at three months (visit 3), which is the end of the intervention treatment period, and at nine months (visit 4). Participants who withdraw from the trial early will not be replaced and will be requested to complete the next scheduled follow-up questionnaire. The schedule of enrolment and data collected during the trial is detailed in Table 4.

Outcomes

Patient-reported outcomes covering pain, function, and health-related quality–of-life will be measured alongside basic clinical findings at baseline and at the three-month (12-week) and nine-month follow-up time points (Figure 1 and Table 4). These include validated outcome measures used with OA patients and in knee OA clinical trials.[35–38] Data on the number of contacts made with hospital and community health services and any costs incurred due to their knee pain (e.g. prescription, travel costs to attend appointments), will be collected at the three and nine-month time points.

Data management and monitoring

Identifiable data will be locked in a filing cabinet in the Leeds Institute of Rheumatic and Musculoskeletal Medicine (LIRMM) research offices or held in an encrypted file stored on a password-protected University of Leeds server, with access limited to the study team. Pseudonymised data will be entered onto a password-protected Access database, developed in line with the LIRMM Data Quality Management System Standard Operating Procedures. Data will be periodically internally verified and audited. Data will be stored in a de-identified manner for 5 years after the final publication.

For peer review only

Table 4: SPIRIT (Standard Protocol Items for Randomized Trials) schedule of enrolment, interventions, and assessments.

	**STUDY PERIOD**			
	**Enrolment**	**Randomisation**	**Follow-Up**	**Close-Out**
**TIMEPOINT**	***-1***	**0**	***3-Months +/- 4 weeks***	***9-Months +/- 4 weeks***
**ENROLMENT:**				
Initial contact & study information	X			
Eligibility screen	X			
Informed consent	X			
Allocation		X		
**INTERVENTIONS:**				
Group E-Rehab			←——→	
'My Knee UK' Group			←——→	
Control Group (duration of treatment will be variable)			←- - - -→	
**ASSESSMENTS:**				
Clinical information				
Age, gender, ethnicity		X		
Previous joint surgery		X		
Employment & physical activity		X		
Height & weight		X		
History of current knee pain		X		
General health & medication use		X	X	X
Patient-reported outcomes				
Joint pain manikin		X	X	X
Knee pain frequency (5-point scale)		X	X	X
Confidence & motivation to do exercises (11-point NRS)		X	X	X
Global change (7-point Likert scale)		X	X	X
WOMAC (Pain, function & stiffness)		X	X	X
ASES		X	X	X
HADS		X	X	X
Generic health status (EQ-5D-5L)		X	X	X
Health-related quality of life (SF-12)		X	X	X
Resource use			X	X

Abbreviations: *NRS: numeric rating scale. WOMAC: The Western Ontario and McMaster Universities Osteoarthritis Index. ASES: Arthritis Self-Efficacy Scale. HADS: Hospital Anxiety and Depression Scale. EQ-5D-5L: European Quality of Life-Five Dimension-Five Level Scale. SF-12: 12-Item Short Form Survey.*

For peer review only - http://bmjopen.bmj.com/site/about/guidelines.xhtml

Physiotherapists will record the time taken to administer and lead the Group E-Rehab exercise classes and if a trial physiotherapist is contacted by a My Knee UK participant for help or advice, this will also be recorded. This data will contribute to estimating the cost of delivering the Group E-Rehab and My Knee UK interventions.

Descriptive data relating to exercise adherence relevant to each treatment arm will be collected at the end of the 12-week intervention phase. The web analytics service Google Analytics[39] will be used to record the number of times each participant accesses the My Knee UK website and the duration of their website access. The weekly number of home-based exercise sessions completed (self-reported in response to the automated SMS message) will also be recorded. Group E-Rehab data will include the number of Zoom exercise classes attended and the number of Sway educational sessions accessed, along with the time spent engaged with each session.

Nested qualitative study

A sub-sample of consenting participants, purposively sampled by age, sex, and having received the My Knee UK or Group E-Rehab intervention, will undergo individual in-depth remote (videoconference or telephone) interviews where the acceptability of the two e-rehabilitation interventions will be explored. Interviews will focus on participants' experiences of being in the trial and will comprise questions specific to each intervention. It is anticipated around 20 individuals will be interviewed with the final number being determined once data saturation has been reached, which will be when there is consensus amongst the research team that minimal new information is being generated. Participants will be interviewed either on completion of the 3-month follow-up questionnaire (visit 3), or at the end of follow-up on completion of the 9-month questionnaire (visit 4).

Semi-structured videoconference interviews with the two physiotherapists who delivered the Group E-Rehab Zoom classes will be conducted. This is to gain insight about the preparation they received,

For peer review only

For peer review only - http://bmjopen.bmj.com/site/about/guidelines.xhtml

thoughts about its acceptability, their experience of delivering the intervention and any barriers to delivering it effectively that they identified.

Planned analyses

The intention-to-treat participant population will be used. A full statistical analysis plan (SAP V1.0, 04/01/2021) was written prior to commencement of recruitment.

Sample size

A total sample size of 90 participants (30 per arm), based on established principles for a feasibility study[40], will be adequate for evaluating feasibility and collecting sufficient data to inform the sample size in a definitive randomised controlled trial (RCT).

Feasibility and proof-of-concept

As a feasibility study, inferential statistics will be limited. Analysis will focus on descriptive statistics, including measures of frequency (e.g. percent), central tendency (e.g. mean, median), and dispersion or variation (e.g. standard deviation, inter-quartile range, confidence interval estimation), rather than formal hypothesis testing. Data will provide an estimate of recruitment and retention rates and the correlation between baseline and follow-up measurements, to inform the sample size required for a definitive RCT. In line with CONSORT[24] the choice of primary outcome for the definitive study will be informed by the results of the feasibility study and guided by a previous study,[10] but candidate variables will be The Western Ontario and McMaster Universities Osteoarthritis Index (WOMAC), pain and physical function domain scores. Published minimum clinically important differences (MCID) reported for the candidate primary outcomes will be used when calculating sample size.[41] If the difference between the groups favours one or both intervention arms over the control arm, and the two-sided 85% confidence interval around the difference includes the MCID, we will proceed to design a definitive trial provided feasibility criteria are met.[42] Attrition will be examined to identify any factors that may be systematically affecting drop-out, and

For peer review only - http://bmjopen.bmj.com/site/about/guidelines.xhtml

continuous measures of adherence within each treatment arm will be summarised. The cost of delivering the e-rehabilitation interventions will be estimated and alongside this, a descriptive report on the use of additional resources (e.g. the type of resources, number of contacts, and any costs incurred) will be produced.

Safety analyses

Adverse events (AEs) or serious adverse events (SAEs) will be recorded and coded to indicate the major event category but, as this is not a clinical trial of an investigational medicinal product, severity will not be graded. The frequency of all treatment-related AEs and SAEs recorded during the trial period will be displayed as the number of participants experiencing the AEs/SAEs, the percentage of participants, and the number of AEs/SAEs will be presented both overall and by treatment arm.

Qualitative study

Data derived from the participant and semi-structured physiotherapist interviews will be transcribed verbatim. Transcripts will be analysed using framework analysis.[43]

Feasibility trial outcome

Data from the feasibility trial and follow-up nested qualitative study will be integrated to support the case for determining whether one or both e-rehabilitation interventions are feasible, acceptable and have the potential to be implemented in practice. The qualitative and quantitative data will enable the potential for My Knee UK and/or Group E-Rehab to be introduced as alternative models of services delivery to be explored. The feasibility trial will be deemed successful if the results demonstrate that (1) participants and physiotherapists find one or both intervention(s) acceptable (using data from the nested qualitative study), (2) it is possible to calculate a manageable sample size for use in a definitive RCT, (3) attrition at visit 3 (3-months) is no more than 30% and (4) at least 40% of eligible patients are recruited to the trial. If one or both e-rehabilitation interventions are

For peer review only

For peer review only - http://bmjopen.bmj.com/site/about/guidelines.xhtml

acceptable, the intention is to develop the protocol for and conduct a definitive RCT with a health economic component. This will be sufficiently powered for testing the wider use of My Knee UK and/or Group E-Rehab (depending on feasibility trial outcome) as a prescribed treatment for individuals with persistent knee pain.

DISCUSSION

The growing prevalence of persistent knee pain and OA requires the development and implementation of effective and accessible treatments that enable these patients to manage their symptoms. These treatments should be convenient for patients and should not overburden local MSK physiotherapy services. Current evidence suggests that for chronic conditions such as OA, digital technologies can be used to deliver self-management programmes electronically.[9,44]

This trial aims to evaluate the feasibility and acceptability of two different e-rehabilitation interventions (Group E-Rehab and My Knee UK) in individuals with persistent knee pain. Education, advice about increasing general physical activity levels, and strengthening exercises that can be tailored to individual needs are pivotal components of both interventions. Muscle strengthening programmes are beneficial in reducing pain and improving physical function in people with knee OA.[45] In this trial, the home-based lower-limb strengthening exercise programme is either prescribed and monitored by a physiotherapist as a group-based intervention using videoconferencing (Group E-Rehab), or it is self-directed (accessed via the My Knee UK website). It is believed that the e-rehabilitation interventions under investigation in this trial will provide patients with access to the digital tools and resources needed for self-managing their knee pain and symptoms, and that one or both interventions could eventually be implemented within the NHS.

For peer review only

For peer review only - http://bmjopen.bmj.com/site/about/guidelines.xhtml

Ethics and dissemination

This feasibility trial and current protocol (v5.0, 25/01/2022) was approved by the West of Scotland Research Ethics Committee 5 (REC5), reference number 20/WS/0006 prior to commencing recruitment, which is ongoing. The study is sponsored by the University of Leeds (Research Integrity and Governance), UK and is registered with the ISRCTN (Reference: 15564385; Supplement 3). All individuals assisting with the trial will be informed of any protocol amendments, which will be approved by the Sponsor before being submitted to the REC/HRA for approval. The results will be disseminated to the study grant funder, submitted for publication in peer-reviewed journals and where requested, a summary of the study findings will be disseminated to study participants. No study participants will be identifiable in the study results.

Data availability statement

The full protocol and statistical analysis plan will be available via the University of Leeds data repository. The full anonymised trial data set and statistical codes will be available 12 months following publication upon request.

For peer review only - http://bmjopen.bmj.com/site/about/guidelines.xhtml

Acknowledgments

The authors would like to thank Alex G Web Design, Melbourne, Australia for duplicating the My Knee Exercise website for use in the UK and SMS Solutions Australia for developing the SMS programme and online interface for use in the UK. The authors would like to thank the clinicians, physiotherapists, patients, PPI members and other experts who provided input into the refinement of the My Knee UK and Group E-Rehab programmes during Phase 1 of the study. The authors would also like to thank the LIRMM clinical trials staff for their help with administering this study.

Competing interests

None declared.

Author contributions

RSH, RKN and KLB co-developed the Australian SMS programme and My Knee Exercise website. Funding was secured by PGC (lead applicant) and GAM, SRK, EMAH, KLB and CC (co-applicants). PGC, SRK, KLB, CC, EMAH, GAM, MC and DGW designed this protocol. EMAH produced the statistical analysis plan. The UK exercise and SMS programmes were developed by PGC, CC, DGW and MC, DGW respectively. DGW designed the UK version of the website (My Knee UK), created the Microsoft Sway education package, is leading the coordination of the study, and drafted the manuscript. All authors contributed to manuscript reviewing and editing and approved the final version.

Funding

This work is being supported by a grant from Versus Arthritis (grant number 22091). This paper presents independent research funded/supported by the National Institute for Health Research (NIHR) Leeds Biomedical Research Centre (BRC). The views expressed are those of the authors and

For peer review only

For peer review only - http://bmjopen.bmj.com/site/about/guidelines.xhtml

not necessarily those of the NIHR or the Department of Health and Social Care. RSH is supported by a National Health and Medical Research Council Senior Research Fellowship (#1154217). The funding sources and sponsor had no role in the study design, writing the manuscript or the decision to submit for publication.

For peer review only

For peer review only - http://bmjopen.bmj.com/site/about/guidelines.xhtml

REFERENCES

1 Thomas E, Peat G, Mallen C, *et al.* Predicting the course of functional limitation among older adults with knee pain: Do local signs, symptoms and radiographs add anything to general indicators? *Ann Rheum Dis* 2008;67:1390–8. doi:10.1136/ard.2007.080945

2 Jinks C, Jordan K, Croft P. Measuring the population impact of knee pain and disability with the Western Ontario and McMaster Universities Osteoarthritis Index (WOMAC). *Pain* 2002;100:55–64. doi:10.1016/S0304-3959(02)00239-7

3 Cui A, Li H, Wang D, *et al.* Global, regional prevalence, incidence and risk factors of knee osteoarthritis in population-based studies. *EClinicalMedicine* 2020;29–30. doi:10.1016/j.eclinm.2020.100587

4 Swain S, Sarmanova A, Mallen C, *et al.* Trends in incidence and prevalence of osteoarthritis in the United Kingdom: findings from the Clinical Practice Research Datalink (CPRD). *Osteoarthr Cartil* 2020;28:792–801. doi:10.1016/j.joca.2020.03.004

5 Arthritis Research UK. Osteoarthritis in General Practice: Data and perspectives. 2013. https://www.versusarthritis.org/media/2115/osteoarthritis-in-general-practice.pdf

6 Conaghan PG, Porcheret M, Kingsbury SR, *et al.* Impact and therapy of osteoarthritis: the Arthritis Care OA Nation 2012 survey. *Clin Rheumatol* 2015;34:1581–8. doi:10.1007/s10067-014-2692-1

7 NICE. Osteoarthritis : care and management (Clinical guideline [CG177]). 2014. https://www.nice.org.uk/guidance/cg177

8 Lawford BJ, Bennell KL, Kasza J, *et al.* Physical Therapists' Perceptions of Telephone- and Internet Video–Mediated Service Models for Exercise Management of People With Osteoarthritis. *Arthritis Care Res* 2018;70:398–408. doi:10.1002/acr.23260

9 Bennell KL, Nelligan R, Dobson F, *et al.* Effectiveness of an internet-delivered exercise and pain-coping skills training intervention for persons with chronic knee pain: A randomized trial. *Ann Intern Med* 2017;166:453–62. doi:10.7326/M16-1714

10 Bennell KL, Lawford BJ, Keating C, *et al.* Comparing Video-Based, Telehealth-Delivered Exercise and Weight Loss Programs With Online Education on Outcomes of Knee Osteoarthritis: A Randomized Trial. *Ann Intern Med* Published Online First: 2021. doi:10.7326/M21-2388

For peer review only

For peer review only - http://bmjopen.bmj.com/site/about/guidelines.xhtml

11 NHS England and NHS Improvement. Clinical guide for the management of remote consultations and remote working in secondary care during the coronavirus pandemic. *NHS Engl* 2020;:1–16.https://www.nice.org.uk/media/default/about/covid-19/specialty-guides/specialty-guide-virtual-working-and-coronavirus.pdf

12 Vusirikala A, Ensor D, Asokan AK, *et al.* Hello, can you hear me? Orthopaedic clinic telephone consultations in the COVID-19 era- a patient and clinician perspective. *World J Orthop* 2021;12:24–34. doi:10.5312/WJO.V12.I1.24

13 Bennell KL, Lawford BJ, Metcalf B, *et al.* Physiotherapists and patients report positive experiences overall with telehealth during the COVID-19 pandemic: a mixed-methods study. *J Physiother* 2021;67:201–9. doi:10.1016/j.jphys.2021.06.009

14 Age UK. Digital inclusion and older people – how have things changed in a Covid-19 world? London: 2021. https://www.ageuk.org.uk/globalassets/age-uk/documents/reports-and-publications/reports-and-briefings/active-communities/digital-inclusion-in-the-pandemic-final-march-2021.pdf (accessed 4 Nov 2021).

15 Nelligan RK, Hinman RS, Kasza J, *et al.* Effects of a Self-directed Web-Based Strengthening Exercise and Physical Activity Program Supported by Automated Text Messages for People with Knee Osteoarthritis: A Randomized Clinical Trial. *JAMA Intern Med* 2021;181:776–85. doi:10.1001/jamainternmed.2021.0991

16 Nelligan RK, Hinman RS, Teo PL, *et al.* Exploring attitudes and experiences of people with knee osteoarthritis toward a self-directed ehealth intervention to support exercise: Qualitative Study. *JMIR Rehabil Assist Technol* 2020;7. doi:10.2196/18860

17 Horton M. CSP: Cuts to physiotherapy services a false economy. Just Physio. 2016;:Online.https://www.justphysio.co.uk/physiotherapy-article-801817162-csp_cuts_to_physiotherapy.html (accessed 22 Jul 2021).

18 Oxtoby K. Covid-19: "Life on hold" for NHS patients needing musculoskeletal care. *BMJ* 2021;373:n1616. doi:10.1136/bmj.n1616

19 Paul J, Hanna JB. Applying the marketing concept in health care: The no-show problem. *Health Mark Q* 1997;14:3–17. doi:10.1300/J026v14n03_02

20 Tan E, Shah A, De Souza W, *et al.* Improving the patient booking service to reduce the number of missed appointments at East London NHS Foundation Trust Community Musculoskeletal Physiotherapy Service. *BMJ Open Qual* 2017;6:e000093. doi:10.1136/BMJOQ-2017-000093

For peer review only

21 Collins J, Santamaria N, Clayton L. Why outpatients fail to attend their scheduled appointments: a prospective comparison of differences between attenders and non-attenders. *Aust Health Rev* 2003;26:52–63. doi:10.1071/AH030052

22 Chan A-W, Tetzlaff JM, Gøtzsche PC, *et al.* SPIRIT 2013 explanation and elaboration: guidance for protocols of clinical trials. *BMJ* 2013;346. doi:10.1136/bmj.e7586

23 Chan A-W, Tetzlaff JM, Altman DG, *et al.* SPIRIT 2013 Statement: Defining Standard Protocol Items for Clinical Trials. *Ann Intern Med* 2013;158:200–7. doi:10.7326/0003-4819-158-3-201302050-00583

24 Eldridge SM, Chan CL, Campbell MJ, *et al.* CONSORT 2010 statement: Extension to randomised pilot and feasibility trials. *Pilot Feasibility Stud* 2016;2. doi:10.1186/s40814-016-0105-8

25 Orkin AM, Gill PJ, Ghersi D, *et al.* Guidelines for Reporting Trial Protocols and Completed Trials Modified Due to the COVID-19 Pandemic and Other Extenuating Circumstances: The CONSERVE 2021 Statement. *JAMA* Published Online First: 21 June 2021. doi:10.1001/jama.2021.9941

26 Jensen MP, Karoly P. Self-report scales and procedures for assessing pain in adults. In: Turk DC, Melzack R, eds. *Handbook of Pain Assessment*. New York: : Guilford Press 2011. 19–44.https://web.s.ebscohost.com/ehost/ebookviewer/ebook/bmxlYmtfXzM0NjE5Ml9fQU41?sid=487b6ed8-a929-49e5-8236-72b3dc113ff7@redis&vid=0&format=EB&lpid=lp_3&rid=0

27 Nelligan RK, Hinman RS, McManus F, *et al.* Moderators of the Effect of a Self-directed Digitally Delivered Exercise Program for People With Knee Osteoarthritis: Exploratory Analysis of a Randomized Controlled Trial. *J Med Internet Res* 2021;23:e30768. doi:10.2196/30768

28 Hurley M, Dickson K, Hallett R, *et al.* Exercise interventions and patient beliefs for people with hip, knee or hip and knee osteoarthritis: A mixed methods review. *Cochrane Database Syst Rev* Published Online First: 2018. doi:10.1002/14651858.CD010842.pub2

29 Abroms LC, Whittaker R, Free C, *et al.* Developing and pretesting a text messaging program for health behavior change: Recommended steps. *JMIR mHealth uHealth* 2015;3. doi:10.2196/mhealth.4917

30 Bennell K, Nelligan RK, Schwartz S, *et al.* Behavior change text messages for home exercise adherence in knee osteoarthritis: Randomized trial. *J Med Internet Res* 2020;22:e21749. doi:10.2196/21749

For peer review only

31 Nelligan RK, Hinman RS, Atkins L, *et al.* A short message service intervention to support adherence to home-based strengthening exercise for people with knee osteoarthritis: Intervention design applying the behavior change wheel. *JMIR mHealth uHealth* 2019;7:1–12. doi:10.2196/14619

32 Jones CJ, Rikli RE, Beam WC. A 30-s chair-stand test as a measure of lower body strength in community-residing older adults. *Res Q Exerc Sport* 1999;70:113–9. doi:10.1080/02701367.1999.10608028

33 Microsoft Corporation. Microsoft Sway [Internet]. 2021.https://sway.office.com (accessed 1 Nov 2021).

34 Sealed Envelope Ltd. Create a blocked randomisation list. 2021.https://www.sealedenvelope.com/simple-randomiser/v1/lists (accessed 18 Jan 2021).

35 Strand V, Kelman A. Outcome measures in osteoarthritis: randomized controlled trials. Curr. Rheumatol. Rep. 2004;6:20–30. doi:10.1007/s11926-004-0080-6

36 Sharma A, Kudesia P, Shi Q, *et al.* Anxiety and depression in patients with osteoarthritis: Impact and management challenges. *Open Access Rheumatol Res Rev* 2016;8:103–13. doi:10.2147/OARRR.S93516

37 McAlindon TE, Driban JB, Henrotin Y, *et al.* OARSI Clinical Trials Recommendations: Design, conduct, and reporting of clinical trials for knee osteoarthritis. *Osteoarthr Cartil* 2015;23:747–60. doi:10.1016/j.joca.2015.03.005

38 Jackson T, Xu T, Jia X. Arthritis self-efficacy beliefs and functioning among osteoarthritis and rheumatoid arthritis patients: A meta-analytic review. *Rheumatol (United Kingdom)* 2020;59:948–58. doi:10.1093/rheumatology/kez219

39 Google.com. About Google Analytics. 2021.https://marketingplatform.google.com/intl/en_uk/about/analytics/ (accessed 1 Nov 2021).

40 Lancaster G, Lancaster GA, Dodd S, *et al.* Correspondence Design and analysis of pilot studies: recommendations for good practice. *J Eval Clin Pract* 2004;10:307–12. doi:10.1111/j..2002.384.doc.x

41 Angst F, Aeschlimann A, Stucki G. Smallest Detectable and Minimal Clinically Important Differences of Rehabilitation Intervention With Their Implications for Required Sample Sizes

For peer review only

Using WOMAC and SF-36 Quality of Life Measurement Instruments in Patients With Osteoarthritis of the Lower Ex. *Arthritis Care Res (Hoboken)* 2001;45:384–91. doi:10.1002/1529-0131(200108)45:4<384::AID-ART352>3.0.CO;2-0

42 Lee EC, Whitehead AL, Jacques RM, *et al.* The statistical interpretation of pilot trials: Should significance thresholds be reconsidered? *BMC Med Res Methodol* 2014;14:1–8. doi:10.1186/1471-2288-14-41

43 Ritchie J, Spencer L. Qualitative data analysis for applied policy research. In: Bryman A, Burgess RG, eds. *Analyzing Qalitative Data*. London: : Routledge 1994. 173–94. doi:10.4324/9780203413081_chapter_9

44 Choi W, Zheng H, Franklin P, *et al.* mHealth technologies for osteoarthritis self-management and treatment: A systematic review. *Health Informatics J* 2019;25:984–1003. doi:10.1177/1460458217735676

45 Fransen M, Mcconnell S, Harmer AR, *et al.* Exercise for osteoarthritis of the knee. Cochrane Database Syst. Rev. 2015;2015. doi:10.1002/14651858.CD004376.pub3

For peer review only

TABLE AND FIGURE TITLES AND LEGENDS

Table 1: Trial eligibility criteria.

Figure 1: Study flow diagram.

Table 2: Summary of the My Knee UK rehabilitation programme.

Table 3: Summary of the Group E-Rehab rehabilitation programme.

Table 4: SPIRIT (Standard Protocol Items for Randomized Trials) schedule of enrolment, interventions, and assessments.

[NRS: numeric rating scale; WOMAC: The Western Ontario and McMaster Universities Osteoarthritis Index; ASES: Arthritis Self-Efficacy Scale; HADS: Hospital Anxiety and Depression Scale; EQ-5D-5L; European Quality of Life-Five Dimension-Five Level Scale; SF-12: 12-Item Short Form Survey]

SUPPLEMENTS

Supplement 1: Protocol modifications made to mitigate the effects of the Covid-19 Pandemic

Supplement 2: Sample participant consent form for the Phase 2 trial

Supplement 3: Trial registration

For peer review only - http://bmjopen.bmj.com/site/about/guidelines.xhtml

EXCLUSIVE LICENCE

I, the Submitting Author has the right to grant and does grant on behalf of all authors of the Work (as defined in the below author licence), an exclusive licence and/or a non-exclusive licence for contributions from authors who are: i) UK Crown employees; ii) where BMJ has agreed a CC-BY licence shall apply, and/or iii) in accordance with the terms applicable for US Federal Government officers or employees acting as part of their official duties; on a worldwide, perpetual, irrevocable, royalty-free basis to BMJ Publishing Group Ltd (“BMJ”) its licensees and where the relevant Journal is co-owned by BMJ to the co-owners of the Journal, to publish the Work in BMJ Open and any other BMJ products and to exploit all rights, as set out in our licence.

The Submitting Author accepts and understands that any supply made under these terms is made by BMJ to the Submitting Author unless you are acting as an employee on behalf of your employer or a postgraduate student of an affiliated institution which is paying any applicable article publishing charge (“APC”) for Open Access articles. Where the Submitting Author wishes to make the Work available on an Open Access basis (and intends to pay the relevant APC), the terms of reuse of such Open Access shall be governed by a Creative Commons licence – details of these licences and which Creative Commons licence will apply to this Work are set out in our licence referred to above.

For peer review only

For peer review only - http://bmjopen.bmj.com/site/about/guidelines.xhtml

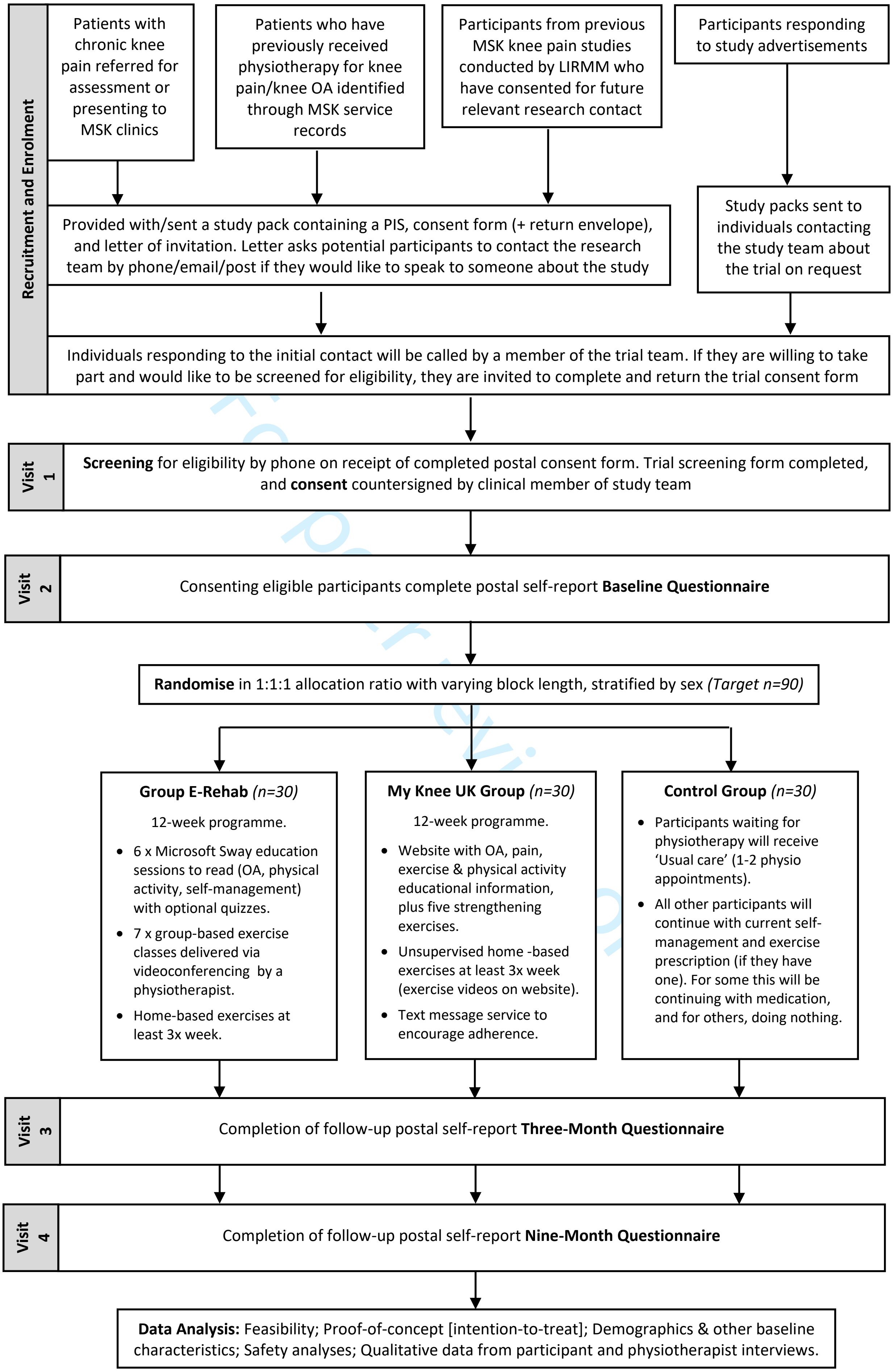

For peer review only - http://bmjopen.bmj.com/site/about/guidelines.xhtml

SUPPLEMENT 1

Protocol modifications made to mitigate the effects of the Covid-19 Pandemic

1. Changes arising from the Covid-19 pandemic mean that routine physiotherapy appointments are now more likely to be remote rather than face-to-face. Currently, most appointments are taking place over the telephone or in some case, via video consultation, rather than being face-to-face in a clinic. Prior to starting the recruitment process for the feasibility trial, the protocol and participant documents were amended to reflect these changes. This means that potential participants will be fully aware that if they enter the study and are allocated to the control group, if they are current patients who will receive usual care, then their physiotherapy appointments are unlikely to be face-to-face, particularly if Covid-19 restrictions are still in place.

2. Given the current and potential future restrictions arising from the Covid-19 pandemic, interviews with the physiotherapists and sub-sample of participants from the trial during the nested qualitative study phase will be done remotely.

3. Current Covid-19 restrictions mean that only skeleton staff members from the research team were physically going into the office. This would have caused a delay in responding to the initial postal response to enquiries from potential participants. To avoid this, the invitation letter was amended slightly so that if anyone receiving the study information is interested in joining the study, they are asked to contact the study team via email or phone instead of returning a paper contact form.

4. A delayed start due to Covid-19 has resulted in shortened timeframe for recruitment, with the risk that reaching the target of 90 participants within the study timeframe would not be achievable. To mitigate this, additional ways of identifying and recruiting participants were added to the protocol. This included:

For peer review only - http://bmjopen.bmj.com/site/about/guidelines.xhtml
For peer review only

- Searching local Trust MSK service records for potentially eligible patients who have previously received physio for knee pain.
- Using Participant identification Centres (PICs) who have the appropriate approval and model agreement in place to carrying out a search of their patient records database to identify individuals that meet a study's eligibility criteria.
- A database search of participants from previous University of Leeds studies who have consented to be contacted about future relevant research studies.
- A media campaign with physical posters/flyers in clinics, waiting rooms or local community centres, and an electronic poster/advert that can be distributed digitally (e.g. email, University or Trust websites), and used via social media.

Participants recruited via MSK waiting lists or clinics (current patients) will continue to receive a physio appointment (usual care) if they are randomised to the control group or if they subsequently withdraw. However, the amendment will affect participants identified via service records, database searches, media campaigns or PICs if they are randomised to the control group or later withdraw from the trial. Participants recruited using these methods (i.e. not via the MSK waiting list or clinics), will be asked to continue with whatever exercises, treatment, or self-management they were doing before joining the trial.

For peer review only

For peer review only - http://bmjopen.bmj.com/site/about/guidelines.xhtml

SUPPLEMENT 2

Sample participant consent form for the Phase 2 trial

UNIVERSITY OF LEEDS

Leeds Institute of Rheumatic and Musculoskeletal Medicine
2nd Floor, Chapel Allerton Hospital
Chapeltown Road, Leeds, LS7 4SA
Email: e.rehab@leeds.ac.uk
Tel: 0113 392 4965

Participant Identification Number

CONSENT FORM (IRAS Reference: 269827)

Evaluation of electronic-rehabilitation programmes for chronic knee pain

Phase 2: Feasibility trial

Please initial each box

1	I confirm that I have read the information sheet dated 18/05/2021 (version 3.0) for the above study. I have had the opportunity to consider the information, ask questions and have had these answered satisfactorily.	
2	I understand that my participation is voluntary and that I am free to withdraw at any time without giving any reason, and without my legal rights or medical care being affected.	
3	I understand that, even if I withdraw from this study, the information already collected from me will be used in analysing the results of this study.	
4	I understand that the group sessions will be recorded and any identifiable audio and visual data from these recordings will be stored securely and not shared.	
5	I understand that the information collected during this study may be used to support other research in the future and may be shared anonymously with other researchers.	
6	I agree to my personal information being stored for the purposes of this study. I understand that any information which could identify me will be kept strictly confidential.	
7	I understand that relevant sections of the data collected during the study, may be looked at by individuals from the University of Leeds (sponsors) or from regulatory authorities where it is relevant to my taking part in this research.	
8	I understand that my GP will be informed about my participation in this study.	
9	I agree to take part in the above study.	

After initialling the boxes, please print and sign your name (participant), add the date, and fill out the participant contact details on the next page before returning the form.

For peer review only - http://bmjopen.bmj.com/site/about/guidelines.xhtml

For peer review only

______________________________ ______________ ________________________
Name of Participant (PRINTED) Date Signature

Please remember to fill in your contact details at the bottom of the page.

The contact slip will be removed and destroyed after we have put your details on to our secure Subject Screening Log, which is used by the research team for contacting you about the study.

______________________________ ______________ ________________________
Name of Researcher *Date Signature

When completed: 1 for participant; 1 for researcher site file. *Will be signed by researcher on a different day.

✂ ....

<u>Participant Contact Details</u>

Telephone Number(s): ____

Email Address: ____

IRAS ID	Project Title	Document Type	Version #	Date	Page
269827	E-rehab for Knee Pain	Phase 2 Trial Patient Consent Form	4.0	10/09/2021	**Page 2 of 2**

For peer review only - http://bmjopen.bmj.com/site/about/guidelines.xhtml

SUPPLEMENT 3

Trial registration

Data category	Information
Registry identifying number	ISRCTN15564385
Date of registration	07/02/2020
Prospective/Retrospective	Prospectively registered
Additional identifiers	CPMS 43473, IRAS 269827
Sources of monetary support	Versus Arthritis; National Institute for Health Research (NIHR) (UK)
Sponsor	University of Leeds
Contact information	Dr Dawn Groves-Williams; d.groves-williams@leeds.ac.uk
	Dr Sarah Kingsbury; s.r.kingsbury@leeds.ac.uk
Short/public title	E-rehab for Knee Pain
Scientific title	Evaluation of electronic-rehabilitation programmes for chronic knee pain
Countries of recruitment	United Kingdom
Condition category	Musculoskeletal Diseases
Condition	Chronic knee pain
Interventions	Access to **My Knee UK** website containing exercise videos and self-management resources for 12 weeks. Support via SMS messages
	**Group E-Rehab**: access to online educational and self-management resources plus 7 online group physiotherapy classes over 12-weeks
	**Control Group:** depending on their recruitment path – will receive usual physiotherapy care or continue with usual self-management
Key inclusion and exclusion criteria	Type: Adults ≥ 18 years; both sexes; no healthy volunteers
	Inclusion: knee pain > 3 months and on most days of previous month; activity-related joint pain; pain during walking ≥4 on an 11-point scale
	Exclusion: Inflammatory arthritis/gout; Joint replacement in study knee; injection within last month; arthroscopy within last 3-months
Study design	Mixed methods interventional randomised controlled feasibility trial with follow-up interviews
	Primary: Interventional; Secondary: Randomised controlled trial; Trial setting: Community; Trial type: treatment
Target sample size	90 (30 in each group)
Date of first enrolment	22/04/2021
Recruitment status	Recruiting
Overall trial status	Ongoing
Key outcomes	Primary: Feasibility and acceptability of the two different electronic-rehabilitation programmes
	Additional: the effect of each electronic-rehabilitation programme on pain and other symptoms compared to the control arm; report any additional resources used by participants during the trial

For peer review only - http://bmjopen.bmj.com/site/about/guidelines.xhtml

Reporting checklist for protocol of a clinical trial.

Based on the SPIRIT guidelines.

Instructions to authors

Complete this checklist by entering the page numbers from your manuscript where readers will find each of the items listed below.

Your article may not currently address all the items on the checklist. Please modify your text to include the missing information. If you are certain that an item does not apply, please write "n/a" and provide a short explanation.

Upload your completed checklist as an extra file when you submit to a journal.

In your methods section, say that you used the SPIRITreporting guidelines, and cite them as:

Chan A-W, Tetzlaff JM, Gøtzsche PC, Altman DG, Mann H, Berlin J, Dickersin K, Hróbjartsson A, Schulz KF, Parulekar WR, Krleža-Jerić K, Laupacis A, Moher D. SPIRIT 2013 Explanation and Elaboration: Guidance for protocols of clinical trials. BMJ. 2013;346:e7586

		Reporting Item	Page Number
**Administrative information**			
Title	#1	Descriptive title identifying the study design, population, interventions, and, if applicable, trial acronym	1
Trial registration	#2a	Trial identifier and registry name. If not yet registered, name of intended registry	4
Trial registration: data set	#2b	All items from the World Health Organization Trial Registration Data Set	Suppl 3
Protocol version	#3	Date and version identifier	21
Funding	#4	Sources and types of financial, material, and other support	23-24
Roles and responsibilities: contributorship	#5a	Names, affiliations, and roles of protocol contributors	1,23

For peer review only

Roles and responsibilities: sponsor contact information	#5b	Name and contact information for the trial sponsor	22
Roles and responsibilities: sponsor and funder	#5c	Role of study sponsor and funders, if any, in study design; collection, management, analysis, and interpretation of data; writing of the report; and the decision to submit the report for publication, including whether they will have ultimate authority over any of these activities	24
Roles and responsibilities: committees	#5d	Composition, roles, and responsibilities of the coordinating centre, steering committee, endpoint adjudication committee, data management team, and other individuals or groups overseeing the trial, if applicable (see Item 21a for data monitoring committee)	8,23
**Introduction**			
Background and rationale	#6a	Description of research question and justification for undertaking the trial, including summary of relevant studies (published and unpublished) examining benefits and harms for each intervention	6-7
Background and rationale: choice of comparators	#6b	Explanation for choice of comparators	7
Objectives	#7	Specific objectives or hypotheses	7
Trial design	#8	Description of trial design including type of trial (eg, parallel group, crossover, factorial, single group), allocation ratio, and framework (eg, superiority, equivalence, non-inferiority, exploratory)	3,7
**Methods: Participants, interventions, and outcomes**			
Study setting	#9	Description of study settings (eg, community clinic, academic hospital) and list of countries where data will	Fg1

For peer review only

		be collected. Reference to where list of study sites can be obtained	
Eligibility criteria	#10	Inclusion and exclusion criteria for participants. If applicable, eligibility criteria for study centres and individuals who will perform the interventions (eg, surgeons, psychotherapists)	Fg1
Interventions: description	#11a	Interventions for each group with sufficient detail to allow replication, including how and when they will be administered	9-15
Interventions: modifications	#11b	Criteria for discontinuing or modifying allocated interventions for a given trial participant (eg, drug dose change in response to harms, participant request, or improving / worsening disease)	N/A
Interventions: adherance	#11c	Strategies to improve adherence to intervention protocols, and any procedures for monitoring adherence (eg, drug tablet return; laboratory tests)	9,12
Interventions: concomitant care	#11d	Relevant concomitant care and interventions that are permitted or prohibited during the trial	8(Tbl1)
Outcomes	#12	Primary, secondary, and other outcomes, including the specific measurement variable (eg, systolic blood pressure), analysis metric (eg, change from baseline, final value, time to event), method of aggregation (eg, median, proportion), and time point for each outcome. Explanation of the clinical relevance of chosen efficacy and harm outcomes is strongly recommended	16-19
Participant timeline	#13	Time schedule of enrolment, interventions (including any run-ins and washouts), assessments, and visits for participants. A schematic diagram is highly recommended (see Figure)	17(Tbl4)
Sample size	#14	Estimated number of participants needed to achieve study objectives and how it was determined, including clinical and statistical assumptions supporting any sample size calculations	19
Recruitment	#15	Strategies for achieving adequate participant enrolment to reach target sample size	8, Fig1

For peer review only

**Methods: Assignment of interventions (for controlled trials)**			
Allocation: sequence generation	#16a	Method of generating the allocation sequence (eg, computer-generated random numbers), and list of any factors for stratification. To reduce predictability of a random sequence, details of any planned restriction (eg, blocking) should be provided in a separate document that is unavailable to those who enrol participants or assign interventions	15
Allocation concealment mechanism	#16b	Mechanism of implementing the allocation sequence (eg, central telephone; sequentially numbered, opaque, sealed envelopes), describing any steps to conceal the sequence until interventions are assigned	15
Allocation: implementation	#16c	Who will generate the allocation sequence, who will enrol participants, and who will assign participants to interventions	15
Blinding (masking)	#17a	Who will be blinded after assignment to interventions (eg, trial participants, care providers, outcome assessors, data analysts), and how	15
Blinding (masking): emergency unblinding	#17b	If blinded, circumstances under which unblinding is permissible, and procedure for revealing a participant's allocated intervention during the trial	N/A
**Methods: Data collection, management, and analysis**			
Data collection plan	#18a	Plans for assessment and collection of outcome, baseline, and other trial data, including any related processes to promote data quality (eg, duplicate measurements, training of assessors) and a description of study instruments (eg, questionnaires, laboratory tests) along with their reliability and validity, if known.	16

For peer review only

		Reference to where data collection forms can be found, if not in the protocol	
Data collection plan: retention	#18b	Plans to promote participant retention and complete follow-up, including list of any outcome data to be collected for participants who discontinue or deviate from intervention protocols	16
Data management	#19	Plans for data entry, coding, security, and storage, including any related processes to promote data quality (eg, double data entry; range checks for data values). Reference to where details of data management procedures can be found, if not in the protocol	16
Statistics: outcomes	#20a	Statistical methods for analysing primary and secondary outcomes. Reference to where other details of the statistical analysis plan can be found, if not in the protocol	19-21
Statistics: additional analyses	#20b	Methods for any additional analyses (eg, subgroup and adjusted analyses)	3,18
Statistics: analysis population and missing data	#20c	Definition of analysis population relating to protocol non-adherence (eg, as randomised analysis), and any statistical methods to handle missing data (eg, multiple imputation)	19
**Methods: Monitoring**			
Data monitoring: formal committee	#21a	Composition of data monitoring committee (DMC); summary of its role and reporting structure; statement of whether it is independent from the sponsor and competing interests; and reference to where further details about its charter can be found, if not in the protocol. Alternatively, an explanation of why a DMC is not needed	N/A (non-CTIMP)
Data monitoring: interim analysis	#21b	Description of any interim analyses and stopping guidelines, including who will have access to these interim results and make the final decision to terminate the trial	N/A
Harms	#22	Plans for collecting, assessing, reporting, and managing solicited and spontaneously reported adverse events	20

For peer review only

		and other unintended effects of trial interventions or trial conduct	
Auditing	#23	Frequency and procedures for auditing trial conduct, if any, and whether the process will be independent from investigators and the sponsor	16
**Ethics and dissemination**			
Research ethics approval	#24	Plans for seeking research ethics committee / institutional review board (REC / IRB) approval	4,22
Protocol amendments	#25	Plans for communicating important protocol modifications (eg, changes to eligibility criteria, outcomes, analyses) to relevant parties (eg, investigators, REC / IRBs, trial participants, trial registries, journals, regulators)	21-22
Consent or assent	#26a	Who will obtain informed consent or assent from potential trial participants or authorised surrogates, and how (see Item 32)	15
Consent or assent: ancillary studies	#26b	Additional consent provisions for collection and use of participant data and biological specimens in ancillary studies, if applicable	N/A
Confidentiality	#27	How personal information about potential and enrolled participants will be collected, shared, and maintained in order to protect confidentiality before, during, and after the trial	16,22
Declaration of interests	#28	Financial and other competing interests for principal investigators for the overall trial and each study site	22
Data access	#29	Statement of who will have access to the final trial dataset, and disclosure of contractual agreements that limit such access for investigators	22
Ancillary and post trial care	#30	Provisions, if any, for ancillary and post-trial care, and for compensation to those who suffer harm from trial participation	N/A
Dissemination policy: trial results	#31a	Plans for investigators and sponsor to communicate trial results to participants, healthcare professionals, the	4, 22

For peer review only

		public, and other relevant groups (eg, via publication, reporting in results databases, or other data sharing arrangements), including any publication restrictions	
Dissemination policy: authorship	#31b	Authorship eligibility guidelines and any intended use of professional writers	31
Dissemination policy: reproducible research	#31c	Plans, if any, for granting public access to the full protocol, participant-level dataset, and statistical code	22
**Appendices**			
Informed consent materials	#32	Model consent form and other related documentation given to participants and authorised surrogates	Suppl 2
Biological specimens	#33	Plans for collection, laboratory evaluation, and storage of biological specimens for genetic or molecular analysis in the current trial and for future use in ancillary studies, if applicable	N/A

None The SPIRIT Explanation and Elaboration paper is distributed under the terms of the Creative Commons Attribution License CC-BY-NC. This checklist can be completed online using https://www.goodreports.org/, a tool made by the EQUATOR Network in collaboration with Penelope.ai

For peer review only

Evaluation of two electronic-rehabilitation programmes for persistent knee pain: protocol for a randomised feasibility trial

Journal:	*BMJ Open*
Manuscript ID	bmjopen-2022-063608.R1
Article Type:	Protocol
Date Submitted by the Author:	03-May-2022

Complete List of Authors:	Groves-Williams, Dawn; University of Leeds Faculty of Medicine and Health, Leeds Institute of Rheumatic and Musculoskeletal Medicine, Chapel Allerton Hospital<br>McHugh, Gretl; University of Leeds School of Healthcare<br>Bennell, Kim; The University of Melbourne Centre for Health Exercise and Sports Medicine, Department of Physiotherapy<br>Comer, Christine; Leeds Community Healthcare NHS Trust, Musculoskeletal and Rehabilitation Service; University of Leeds Faculty of Medicine and Health, Leeds Institute of Rheumatic and Musculoskeletal Medicine, Chapel Allerton Hospital<br>Hensor, Liz; University of Leeds Faculty of Medicine and Health, Leeds Institute of Rheumatic and Musculoskeletal Medicine, Chapel Allerton Hospital; NIHR Leeds Biomedical Research Centre<br>Conner, Mark; University of Leeds School of Psychology<br>Nelligan, Rachel; The University of Melbourne Centre for Health Exercise and Sports Medicine, Department of Physiotherapy<br>Hinman, Rana; The University of Melbourne Centre for Health Exercise and Sports Medicine, Department of Physiotherapy<br>Kingsbury, Sarah; University of Leeds Faculty of Medicine and Health, Leeds Institute of Rheumatic and Musculoskeletal Medicine, Chapel Allerton Hospital; NIHR Leeds Biomedical Research Centre,<br>Conaghan, Philip; University of Leeds Faculty of Medicine and Health, Leeds Institute of Rheumatic and Musculoskeletal Medicine, Chapel Allerton Hospital; NIHR Leeds Biomedical Research Centre

**Primary Subject Heading**:	Rheumatology
Secondary Subject Heading:	Sports and exercise medicine
Keywords:	PAIN MANAGEMENT, Rheumatology < INTERNAL MEDICINE, World Wide Web technology < BIOTECHNOLOGY & BIOINFORMATICS, Telemedicine < BIOTECHNOLOGY & BIOINFORMATICS

SCHOLARONE™
Manuscripts

Evaluation of two electronic-rehabilitation programmes for persistent knee pain: protocol for a randomised feasibility trial

Dawn Groves-Williams [a], Gretl A. McHugh [c], Kim L. Bennell [d], Christine Comer [e,a], Elizabeth M A Hensor[a,b], Mark Conner [f], Rachel K. Nelligan [d], Rana S Hinman [d], Sarah R. Kingsbury [a,b,*], Philip G. Conaghan [a,b,*]

Corresponding author.

Assoc Prof Sarah Kingsbury, Leeds Institute of Rheumatic and Musculoskeletal Medicine (LIRMM), 2nd Floor, Chapel Allerton Hospital, Chapeltown Road, Leeds LS7 4SA, UK.

Email: s.r.kingsbury@leeds.ac.uk; Telephone: +44 113 392 4883

Author affiliations

[a] Leeds Institute of Rheumatic and Musculoskeletal Medicine (LIRMM), School of Medicine, Chapel Allerton Hospital, Leeds.

[b] NIHR Leeds Biomedical Research Centre (BRC), Chapel Allerton Hospital, Leeds, UK.

[c] School of Healthcare, University of Leeds, Leeds, UK.

[d] Centre for Health Exercise and Sports Medicine, Department of Physiotherapy, School of Health Sciences, The University of Melbourne, Melbourne, Australia.

[e] Leeds Community Healthcare NHS Trust, Wetherby Health Centre, Leeds, UK.

[f] School of Psychology, University of Leeds, Leeds, UK.

[*]These authors contributed equally.

KEYWORDS

Pain management

Rheumatology

For peer review only

For peer review only - http://bmjopen.bmj.com/site/about/guidelines.xhtml

World wide web technology

Telemedicine

WORD COUNT

Abstract: 298

Manuscript: 3800 (excl. title page, abstract, table contents, references)

For peer review only

ABSTRACT

Introduction

Persistent, knee pain is a common cause of disability. Education and exercise treatment are advocated in all clinical guidelines; however, the increasing prevalence of persistent knee pain presents challenges for health services regarding appropriate and scalable delivery of these treatments. Digital technologies may help address this, and this trial will evaluate the feasibility and acceptability of two electronic-rehabilitation (e-rehabilitation) interventions: 'My Knee UK' and 'Group E-Rehab'.

Methods and analysis

This protocol describes a non-blinded, randomised feasibility trial with three parallel groups. The trial aims to recruit 90 participants (45 years or older) with a history of persistent knee pain consistent with a clinical diagnosis of knee osteoarthritis (OA). Participants will be randomly assigned in a 1:1:1 allocation ratio. The 'My Knee UK' intervention arm will receive a self-directed unsupervised Internet-based home exercise programme plus short message service (SMS) support (targeting exercise behaviour change) for 12-weeks; the 'Group E-Rehab' intervention arm will receive group-based physiotherapist-prescribed home exercises delivered via videoconferencing accompanied by Internet-interactive educational sessions for 12-weeks; the control arm will receive usual physiotherapy care or continue with their usual self-management (depending on their recruitment path). Feasibility variables, patient-reported outcomes and clinical findings measured at baseline, three and nine-months will be assessed and integrated with qualitative interview data from a sub-set of Group E-Rehab and My Knee UK participants. If considered feasible and acceptable, a definitive randomised controlled trial can be conducted to investigate the clinical- and cost-effectiveness of one or both interventions with a view to implementation in routine care.

For peer review only - http://bmjopen.bmj.com/site/about/guidelines.xhtml

Ethics and dissemination

The trial was approved by the West of Scotland Research Ethics Committee 5 (Reference: 20/WS/0006). The results of the study will be disseminated to study participants, the study grant funder, and will be submitted for publication in peer-reviewed journals.

Trial registration number

ISRCTN: 15564385.

For peer review only

For peer review only - http://bmjopen.bmj.com/site/about/guidelines.xhtml

ARTICLE SUMMARY

Strengths and limitations of this study

- This study does not require any face-to-face contact as the interventions are accessed and/or delivered remotely.
- The digital health interventions being investigated have been tailored to the needs of individuals with persistent knee pain based on feedback from patient and public involvement and expert review groups conducted during Phase 1.
- Data will be collected and analysed using a mixed methods approach to provide a greater understanding of the feasibility and acceptability of the two digital health interventions.
- Participants, physiotherapists, and the trials staff administering the study cannot be blinded, however, the trial statistician will remain blinded to group allocation until the database has been locked.
- The interventions require participants to have an active email account, mobile phone, and access to a computer or tablet with Internet access suitable for receiving/making video calls.

For peer review only

INTRODUCTION

Persistent or chronic knee pain, often with associated stiffness and functional limitations, is a common problem in older and middle-aged adults.[1,2] A leading cause is osteoarthritis (OA) and in 2020, the pooled global prevalence of knee OA in individuals aged 40 and over was reported to be around 23%, with a positive correlation between prevalence and increased age.[3] Approximately 10% of adults in the United Kingdom (UK) have a clinical diagnosis of OA, with symptomatic knee OA being the commonest site.[4] It is estimated that by 2035 the number of people with knee OA in the UK could reach 8.3 million.[5] Exercise and access to appropriate education are advocated in all clinical guidelines as core treatments for persistent knee pain. However, with the increasing prevalence of persistent knee pain and current treatment delivery strategies not always addressing patient needs, managing these patients is challenging.[6]

Physiotherapists are key in providing education, exercises, and self-management support to improve symptoms and function for individuals with knee OA.[7] It has previously been observed that physiotherapists can effectively deliver OA rehabilitation interventions remotely using video technologies,[8–10] which is increasingly important given the adoption of telehealth during the Covid-19 pandemic[11–13]. It has been reported that since the onset of Covid-19, 88% of 50-64 year olds, 75% of 65-74 year olds and 46% of those aged 75 or over use the Internet almost every day or every day.[14] These data support the assumption that some, but not all older individuals could access Internet-based electronic interventions.

Digital health interventions for knee pain have been developed and delivered by physiotherapists in Australia; and there is evidence to suggest they can improve knee pain and function[9,15] and are generally accepted by patients with knee OA.[16] Such digital health interventions may be useful in other large health services (such as in the UK) as an alternative to “in person” clinic appointments, particularly where physiotherapy services are overstretched[17] and waiting times are prolonged

For peer review only - http://bmjopen.bmj.com/site/about/guidelines.xhtml

due to the Covid-19 pandemic,[18] or where individuals are unable to attend physiotherapy sessions due to mobility problems and/or lack of transport.[19–21]

Aims and objectives

This trial aims to evaluate the feasibility and acceptability of two different electronic-rehabilitation (e-rehabilitation) interventions in individuals with persistent knee pain. One comprises a self-directed Internet-based home exercise programme with exercise behaviour change support provided via automated SMS ('My Knee UK'). The second is a group-based home exercise programme with Internet-interactive education sessions ('Group E-Rehab'), where the home exercise programme is prescribed and monitored by a physiotherapist using videoconferencing. The trial will also explore the effect of each e-rehabilitation programme on pain and other symptoms compared to the control arm and provide a report on any additional resources (e.g. additional NHS/private healthcare services) used by participants during the trial.

METHODS

Trial design

This is an unblinded, single-centre, randomised feasibility trial with three parallel arms (two e-rehabilitation treatment arms and one control arm). The study protocol was designed to conform to the Standard Protocol Items: Recommendations for Interventional Trials (SPIRIT) guidelines[22,23] and the extension of the Consolidated Standards of Reporting Trials (CONSORT) statement for randomised pilot and feasibility trials.[24] The feasibility trial (Phase 2) opened to recruitment in March 2021 and the planned study end date is June 2023. Trial phases are outlined in Figure 1.

*(Insert **Figure 1:** Study flow diagram.)*

For peer review only - http://bmjopen.bmj.com/site/about/guidelines.xhtml

136 In line with the recently published CONSERVE 2021 Statement and guidelines,[25] several protocol changes were made in response to the Covid-19 pandemic (Supplement 1).

Patient and Public Involvement

Members of the NIHR Leeds Biomedical Research Centre Patient and Public Involvement (PPI) group provided input into the trial design and outcome measures through focus group discussions during the funding application stages. PPI was subsequently used to provide input into the refinement of the two e-rehabilitation programmes during the first phase of the study. Additionally, two PPI representatives are members of the Project Advisory Group and help review content and assist with key decisions throughout the trial.

Participants

The target population is adults with persistent knee pain who meet the eligibility criteria (Table 1). Potential participants have either been referred to musculoskeletal (MSK) services for assessment or have previously received physiotherapy for persistent knee pain; are participants from past studies that have consented to future contact; have responded to a media campaign advertising for volunteers (Figure 1).

Table 1: Trial eligibility criteria.

Inclusion Criteria	Exclusion Criteria
Adults ≥ 45 years	Inflammatory arthritis (including gout)
Knee pain > 3 months and on most days of previous month	A joint replacement in the study knee
Knee pain during walking ≥4 on an 11-point numerical rating scale[26]	An injection into the study knee joint within the last month
Activity-related knee joint pain	An arthroscopy of the study knee joint in the last three months
Has a mobile phone, active email account, and computer with Internet access suitable for receiving and making video calls if required.	Enrolled in another research study involving an intervention for OA treatment or management
	Unable to comply with the study protocol
	Unable to understand written and spoken English.

For peer review only - http://bmjopen.bmj.com/site/about/guidelines.xhtml

Digital health intervention treatment arms

The e-rehabilitation interventions were adapted for use in the UK (My Knee UK) or developed (Group E-Rehab) during Phase 1 of the study. Feedback relating to usability and content, gathered using online think-aloud interviews and expert review groups, was used to refine the programmes prior to starting the trial. The results from Phase 1, which supports the robustness of the development of intervention content, will be published separately.

My Knee UK

This is a 12-week Internet-based home exercise intervention that was modified from 'My Knee Exercise' (https://mykneeexercise.org.au), a 24-week intervention created and trialled in Australia by Nelligan et al.[15,27] This intervention provides guidance, via a website, for participants to undertake an unsupervised, self-directed lower limb strengthening programme supported by online OA educational information and advice (Table 2). The strengthening programme was reduced from the 24-week Australian version to what was considered a less burdensome 12-week programme by clinical members of the research team.[28] The exercise programme was reviewed and refined by research team and PPI members of the Project Advisory group, with input from an MSK physiotherapist expert review group.

In this trial, three muscle strengthening exercises are introduced in Programme One (weeks 1-6), and two additional strengthening exercises are added in Programme Two (weeks 7-12). The website (My Knee UK) encourages participants to perform their unsupervised home-based exercises at least three times a week and gives instructions about tailoring and progressing each exercise to their own ability/needs. Exercise logbooks and physical activity planners are available to download from the website to print or complete electronically. Participants are encouraged to access the website at leisure throughout the 12-week intervention and can phone/email a trial physiotherapist if they have any concerns or experience difficulties with the exercise programme.

For peer review only - http://bmjopen.bmj.com/site/about/guidelines.xhtml

Participants receive exercise-related behaviour change messages throughout the My Knee UK intervention via automated SMS delivered to their mobile phones (SMS Solutions Australia, Melbourne, Australia). The SMS library uses behaviour change theory to identify and address key barriers to and facilitators of home-exercise programme adherence[29] and has been shown to increase adherence to unsupervised home-based strengthening exercises.[30] The 24-week SMS script, developed by Nelligan et al.[31] with input from academics, physiotherapists, and consumers, was modified for use with the 12-week My Knee UK intervention by increasing the frequency of facilitator messages (from between 0 & 2 to between 1 & 3 per week), enabling the full BCT message library to be used (Table 2).

For peer review only

For peer review only - http://bmjopen.bmj.com/site/about/guidelines.xhtml

Table 2: Summary of the My Knee UK rehabilitation programme.

Webpage Tab	Contents
**1. Home**	Introductory video (from Prof Conaghan)<br>How to use the website and beginning your programme<br>'Contact Us' for help tab
**2. My Knee Education**	2.1. My knee education introduction<br>2.2. Understanding knee osteoarthritis (OA)<br>2.3. Understanding knee pain<br>2.4. Knee pain treatments<br>2.5. Exercise as treatment<br>2.6. Recommended exercise<br>2.7. Managing exercise pain<br>2.8. How to start in the exercise
**3. My Knee Strength**	3.1. My knee strength introduction<br>3.2. How to start your knee exercises<br>3.3. Organise your exercise equipment<br>3.4. Tips for starting and sticking to exercise<br>3.5. Your mobile phone text message support<br>**Exercise Programme One (weeks 1-6)**<br>Exercise instructions and videos (incl. self-tailoring exercise guides)<br>a) Sitting knee extension, b) Side steps, c) Calf raises.<br>**Exercise Programme One (weeks 2-7)**<br>Exercise instructions and videos (incl. self-tailoring exercise guides)<br>a) Sitting knee extension, b) Side steps, c) Calf raises,<br>d) Mini (wall) squats, e) Chair rises (sit-to-stands).
**4. My Knee Activity**	4.1. My knee activity introduction<br>4.2. Why increase physical activity?<br>4.3. How to increase physical activity<br>4.4. Track your daily steps<br>4.5. Activity pacing<br>4.6. Make a physical activity plan<br>4.7. Record your progress<br>4.8. Physical activity success stories (videos)
**5. My Knee Tools**	Contains all the resources used throughout the website in one place.

**Examples of facilitator and barrier behaviour change messages**[30,31]		
**Facilitator**	Hi [name], fitting in regular knee exercise is hard. The reason we are recommending weekly exercise is because exercise works best when it's a regular thing. Exercising longer term can lead to lasting benefits in your knee health.	Do you have a goal you'd like to achieve if your knee improved? Think about what your goal is. Achieving your knee goals is the reward for doing the exercise programme.
**Barrier**	[Name] It's okay to pull back the intensity of exercises if you're feeling concerned. The important thing is that you do the exercises regularly. Gradually build up again as the knee becomes more stable & your confidence increases.	[Name] It can be hard to remember. We suggest making the exercises a habit. Set aside the same time each day to do them. It's much harder to forget when something is a daily routine.

For peer review only

For peer review only - http://bmjopen.bmj.com/site/about/guidelines.xhtml

Group E-Rehab

Group E-Rehab is a 12-week e-rehabilitation intervention comprising six Internet-interactive education sessions and the same five lower limb strengthening exercises as My Knee UK. However, unlike those allocated to My Knee UK, Group E-Rehab participants receive seven group-based exercise sessions delivered remotely by a physiotherapist (Table 3) via the videoconferencing platform Zoom (Zoom Video Communications Inc., San Jose, USA) in weeks 1, 2, 3, 5, 7, 9, and 12 (Table 3). The physiotherapist demonstrates/teaches the leg strengthening exercises (limited to three exercises in the first three classes), and conducts a 30-second chair sit-to-stand test[32] remotely at the start of each class as a baseline measure and indication of progress. The physiotherapist monitors the sit-to-stand assessment alongside exercise quality, technique, and effort during the classes, and uses these measures to tailor the exercises to meet each participant's needs. Group sessions last 45-60 minutes, and each group contains four-seven participants.

Interactive educational sessions developed for the Group E-Rehab intervention, available through the digital presentation programme Microsoft Sway,[33] cover knee OA self-management and include optional quizzes with automated feedback, plus self-assessment questionnaires to make the sessions more personalised (Table 3). Participants are advised that, although they can access the Sway education sessions in any order and at their leisure during the 12-week intervention, working through all six in the first 6-weeks will give them more opportunities to ask questions and/or discuss the contents during the online exercise classes. The physiotherapist provides dedicated time for this during each online class as well as reminding participants to engage with the education sessions and encouraging them to do their exercises at least three times each week. Participants are emailed exercise logbooks and physical activity planners to download.

Two Leeds Community MSK and Rehabilitation Service physiotherapists with current registration to practice in the UK were identified to deliver the Group E-Rehab intervention based on their clinical skills and experience. In preparation for the trial, both attended an external one-day motivational

For peer review only - http://bmjopen.bmj.com/site/about/guidelines.xhtml

interviewing training course (Et al Training, Leeds, UK). To facilitate consistent delivery of the intervention, particularly the exercise component, the physiotherapists completed practice Zoom classes and follow a comprehensive guidance document (written and provided by the study Advanced Practice Physiotherapist [CC] and DGW).

For peer review only

For peer review only - http://bmjopen.bmj.com/site/about/guidelines.xhtml

Table 3: Summary of the Group E-Rehab rehabilitation programme.

**Sway Session 1: Pain and the Knee Joint**<br>1.1 About the Knee joint<br>Anatomy and physiology / Using the knee joint<br>1.2 What is Pain<br>1.3 What causes knee pain<br>Knee Osteoarthritis (OA) / Some facts about OA<br>1.4 The Pain cycle<br>1.5 Pain Management<br>Activity / Conventional medicine<br>Complementary and alternative medicine<br>Other ways of managing knee pain<br>1.6 Test your knowledge (quiz)	**Sway Session 2: Physical Activity**<br>2.1 The Benefits of Exercise and Physical Activity<br>Reducing the risk of falling<br>How physically active am I? (quiz)<br>2.2 Building an active lifestyle<br>Aerobic and cardiovascular exercise<br>Physical Activity Recommendations<br>2.3 Exercise, general physical activity, and Joint Pain<br>Planning and recording physical activity<br>Tracking your daily steps<br>2.4 The Group E-Rehab Home Exercise Programme<br>Format of the sessions / equipment<br>Leg strengthening programme<br>2.5 Will I get New Aches and Pains if I exercise?<br>2.6 Physical activity quiz
**Sway Session 3: Goal Setting**<br>3.1 Do my knee symptoms hold me back (quiz)<br>3.2 What are goals<br>3.3 Why and How should I set goals?<br>3.4 SMART goals<br>Example of a SMART goal<br>3.5 Goal Planning and Implementation<br>3.6 Problem Solving<br>Problem solving example<br>3.7 Reward yourself<br>3.8 Goal setting and chronic pain (quiz)	**Sway Session 4: Pacing Skills**<br>4.1 Activity levels and Pain<br>4.2 Flare ups<br>4.3 Balancing Activity and Rest<br>Boom Bust (Overactivity/underactivity) cycle<br>Activity rest cycle<br>4.4 Pacing your activity levels<br>Putting Pacing into practice<br>4.5 Pacing and chronic pain quiz (quiz)<br>4.6 Managing at Work
**Sway Session 5: Communication and Emotional Wellbeing**<br>5.1 How well do I communicate with people? (quiz)<br>5.2 The importance of Effective Communication<br>Making others aware (inc. professionals)<br>5.3 Communication Styles<br>Assertive Communication<br>5.5 The Importance of Emotional Wellbeing<br>5.6 Managing Emotions, Acceptance, and Feeling Positive<br>Getting and Staying Connected<br>Distraction techniques / Mindfulness<br>5.7 Managing Setbacks<br>5.8 Emotions and Chronic Pain (quiz)<br>5.9 Online Arthritis Support	**Sway Session 6: Staying Healthy**<br>6.1 Good Health<br>6.2 Healthy Eating<br>6.3 Getting Enough Sleep<br>Sleep and Pain / Sleeping well<br>6.4 Relax and Unwind<br>6.5 Good Health Quiz (quiz)<br>6.6 Summary<br>6.7 What Happens Next?<br>Continuing exercises and activities
**Home-based Leg Strengthening Exercises**<br>**Physiotherapist-led group-based exercise classes via Zoom in weeks 1, 2, 3, 5, 7, 9, and 12.**<br>Weeks 1-6 (3 exercises): a) Sitting knee extension, b) Side steps, c) Calf raises (weeks 1-6).<br>Weeks 7-12 (5 exercises): Exercises a to c plus - d) Mini (wall) squats, e) Chair rises (sit-to-stands).<br>• 30 second sit-to-stand test done at the start of every physio-led Zoom class.<br>• Classes include time for discussing the self-directed Sway educational sessions.	

For peer review only

For peer review only - http://bmjopen.bmj.com/site/about/guidelines.xhtml

Control arm

Participants allocated to the control group could receive any usual care intervention, ranging from no healthcare practitioner input to one or two physiotherapy sessions (delivered in-person or remotely by telephone or videoconferencing), to advice and guidance about self-management. The type of intervention that control group participants receive depends on their recruitment path.

Enrolment

Participants are enrolled once they have been screened for eligibility by telephone (visit 1) and their informed, written postal consent has been countersigned by a delegated member of the research team (Supplement 2). Eligible consenting participants complete a postal baseline questionnaire (visit 2) prior to randomisation.

Randomisation and blinding

Participants are randomised (by DGW) to one of the three groups using a 1:1:1 allocation ratio. Random allocation sequences with varying block length (3, 6 and 9), stratified by sex, are generated using an external password-protected web-based randomisation system (Sealed Envelope Ltd. London, UK). A dummy randomisation list[34], created using the same settings but different random seed number, was set up and then checked by the trial statistician (EMAH) before the trial randomisation list was created.

The nature of this feasibility trial means it is not possible to blind participants, physiotherapists delivering the Group E-Rehab intervention, or the study team managing the trial. However, the trial statistician will remain blinded to group allocation until all preliminary data checks have been performed at a blinded data review meeting and the database has been locked.

For peer review only - http://bmjopen.bmj.com/site/about/guidelines.xhtml

Trial data collection and outcomes

Follow-up visits

Follow-up postal questionnaires are sent out at three months (visit 3), which is the end of the intervention treatment period, and at nine months (visit 4). Participants who withdraw from the trial early will not be replaced and will be requested to complete the next scheduled follow-up questionnaire. The schedule of enrolment and data collected during the trial is detailed in Table 4.

Outcomes

Patient-reported outcomes covering pain, function, health-related quality–of-life, coping and catastrophising, and confidence and motivation to do exercises will be measured alongside basic clinical findings at baseline and at the three-month (12-week) and nine-month follow-up time points (Figure 1 and Table 4). These include validated outcome measures used with OA patients and in knee OA clinical trials.[35–38] Global change in overall pain and mobility/function and data on the number of contacts made with hospital and community health services, plus any costs incurred due to their knee pain (e.g. prescription, travel costs to attend appointments), will be collected at the three and nine-month time points. Primary and secondary outcomes have not been specified as this is a feasibility study.

Data management and monitoring

Identifiable data will be locked in a filing cabinet in the Leeds Institute of Rheumatic and Musculoskeletal Medicine (LIRMM) research offices or held in an encrypted file stored on a password-protected University of Leeds server, with access limited to the study team. Pseudonymised data will be entered onto a password-protected Access database, developed in line with the LIRMM Data Quality Management System Standard Operating Procedures. Data will be periodically internally verified and audited. Data will be stored in a de-identified manner for 5 years after the final publication.

For peer review only - http://bmjopen.bmj.com/site/about/guidelines.xhtml
For peer review only

Table 4: SPIRIT (Standard Protocol Items for Randomized Trials) schedule of enrolment, interventions, and assessments.

	**STUDY PERIOD**			
	**Enrolment**	**Randomisation**	**Follow-Up**	**Close-Out**
**TIMEPOINT**	***-1***	**0**	***3-Months +/- 4 weeks***	***9-Months +/- 4 weeks***
**ENROLMENT:**				
Initial contact & study information	X			
Eligibility screen	X			
Informed consent	X			
Allocation		X		
**INTERVENTIONS:**				
Group E-Rehab			←——→	
'My Knee UK' Group			←——→	
Control Group (duration of treatment will be variable)			←- - - -→	
**ASSESSMENTS:**				
Clinical information				
Age, gender, ethnicity		X		
Previous joint surgery		X		
Employment & physical activity		X		
Height & weight		X		
History of current knee pain		X		
General health & medication use		X	X	X
Patient-reported outcomes				
Joint pain manikin		X	X	X
Knee pain frequency (5-point scale)		X	X	X
Confidence & motivation to do exercises (11-point NRS)		X	X	X
Global change (7-point Likert scale)			X	X
WOMAC (Pain, function & stiffness)		X	X	X
ASES		X	X	X
HADS		X	X	X
Generic health status (EQ-5D-5L)		X	X	X
Health-related quality of life (SF-12)		X	X	X
Resource use			X	X

Abbreviations: *NRS: numeric rating scale. WOMAC: The Western Ontario and McMaster Universities Osteoarthritis Index. ASES: Arthritis Self-Efficacy Scale. HADS: Hospital Anxiety and Depression Scale. EQ-5D-5L: European Quality of Life-Five Dimension-Five Level Scale. SF-12: 12-Item Short Form Survey.*

For peer review only

For peer review only - http://bmjopen.bmj.com/site/about/guidelines.xhtml

Physiotherapists will record the time taken to administer and lead the Group E-Rehab exercise classes and if a trial physiotherapist is contacted by a My Knee UK participant for help or advice, this will also be recorded. This data will contribute to estimating the cost of delivering the Group E-Rehab and My Knee UK interventions.

Descriptive data relating to exercise adherence relevant to each treatment arm will be collected at the end of the 12-week intervention phase. The web analytics service Google Analytics[39] will be used to record the number of times each participant accesses the My Knee UK website and the duration of their website access. The weekly number of home-based exercise sessions completed (self-reported in response to the automated SMS message) will also be recorded. Group E-Rehab data will include the number of Zoom exercise classes attended and the number of Sway educational sessions accessed, along with the time spent engaged with each session.

Nested qualitative study

A sub-sample of consenting participants, purposively sampled by age, sex, and having received the My Knee UK or Group E-Rehab intervention, will undergo individual in-depth remote (videoconference or telephone) interviews where the acceptability of the two e-rehabilitation interventions will be explored. Interviews will focus on participants' experiences of being in the trial and will comprise questions specific to each intervention. It is anticipated around 20 individuals will be interviewed with the final number being determined once data saturation has been reached, which will be when there is consensus amongst the research team that minimal new information is being generated. Participants will be interviewed either on completion of the 3-month follow-up questionnaire (visit 3), or at the end of follow-up on completion of the 9-month questionnaire (visit 4).

Semi-structured videoconference interviews with the two physiotherapists who delivered the Group E-Rehab Zoom classes will be conducted. This is to gain insight about the preparation they received,

For peer review only - http://bmjopen.bmj.com/site/about/guidelines.xhtml

thoughts about its acceptability, their experience of delivering the intervention and any barriers to delivering it effectively that they identified.

Planned analyses

The intention-to-treat participant population will be used. A full statistical analysis plan (SAP V1.0, 04/01/2021) was written prior to commencement of recruitment.

Sample size

A total sample size of 90 participants (30 per arm), based on established principles for a feasibility study[40], will be adequate for evaluating feasibility and collecting sufficient data to inform the sample size in a definitive randomised controlled trial (RCT).

Feasibility and proof-of-concept

As a feasibility study, inferential statistics will be limited. Analysis will focus on descriptive statistics, including measures of frequency (e.g. percent), central tendency (e.g. mean, median), and dispersion or variation (e.g. standard deviation, inter-quartile range, confidence interval estimation), rather than formal hypothesis testing. Data will provide an estimate of recruitment and retention rates and the correlation between baseline and follow-up measurements, to inform the sample size required for a definitive RCT. In line with CONSORT[24] the choice of primary outcome for the definitive study will be informed by the results of the feasibility study and guided by a previous study,[10] but candidate variables will be The Western Ontario and McMaster Universities Osteoarthritis Index (WOMAC), pain and physical function domain scores. Published minimum clinically important differences (MCID) reported for the candidate primary outcomes will be used when calculating sample size.[41] If the difference between the groups favours one or both intervention arms over the control arm, and the two-sided 85% confidence interval around the difference includes the MCID, we will proceed to design a definitive trial provided feasibility criteria are met.[42] Attrition will be examined to identify any factors that may be systematically affecting drop-out, and

For peer review only - http://bmjopen.bmj.com/site/about/guidelines.xhtml

For peer review only

continuous measures of adherence within each treatment arm will be summarised. The cost of delivering the e-rehabilitation interventions will be estimated and alongside this, a descriptive report on the use of additional resources (e.g. the type of resources, number of contacts, and any costs incurred) will be produced.

Safety analyses

Adverse events (AEs) or serious adverse events (SAEs) will be recorded and coded to indicate the major event category but, as this is not a clinical trial of an investigational medicinal product, severity will not be graded. The frequency of all treatment-related AEs and SAEs recorded during the trial period will be displayed as the number of participants experiencing the AEs/SAEs, the percentage of participants, and the number of AEs/SAEs will be presented both overall and by treatment arm.

Qualitative study

Data derived from the participant and semi-structured physiotherapist interviews will be transcribed verbatim. Transcripts will be analysed using framework analysis.[43]

Feasibility trial outcome

Data from the feasibility trial and follow-up nested qualitative study will be integrated to support the case for determining whether one or both e-rehabilitation interventions are feasible, acceptable and have the potential to be implemented in practice. The qualitative and quantitative data will enable the potential for My Knee UK and/or Group E-Rehab to be introduced as alternative models of services delivery to be explored. The feasibility trial will be deemed successful if the results demonstrate that (1) participants and physiotherapists find one or both intervention(s) acceptable (using data from the nested qualitative study), (2) it is possible to calculate a manageable sample size for use in a definitive RCT, (3) attrition at visit 3 (3-months) is no more than 30% and (4) at least 40% of eligible patients are recruited to the trial. If one or both e-rehabilitation interventions are

For peer review only - http://bmjopen.bmj.com/site/about/guidelines.xhtml

acceptable, the intention is to develop the protocol for and conduct a definitive RCT with a health economic component. This will be sufficiently powered for testing the wider use of My Knee UK and/or Group E-Rehab (depending on feasibility trial outcome) as a prescribed treatment for individuals with persistent knee pain.

DISCUSSION

The growing prevalence of persistent knee pain and OA requires the development and implementation of effective and accessible treatments that enable these patients to manage their symptoms. These treatments should be convenient for patients and should not overburden local MSK physiotherapy services. Current evidence suggests that for chronic conditions such as OA, digital technologies can be used to deliver self-management programmes electronically.[9,44]

This trial aims to evaluate the feasibility and acceptability of two different e-rehabilitation interventions (Group E-Rehab and My Knee UK) in individuals with persistent knee pain. Education, advice about increasing general physical activity levels, and strengthening exercises that can be tailored to individual needs are pivotal components of both interventions. Muscle strengthening programmes are beneficial in reducing pain and improving physical function in people with knee OA.[45] In this trial, the home-based lower-limb strengthening exercise programme is either prescribed and monitored by a physiotherapist as a group-based intervention using videoconferencing (Group E-Rehab), or it is self-directed (accessed via the My Knee UK website). It is believed that the e-rehabilitation interventions under investigation in this trial will provide patients with access to the digital tools and resources needed for self-managing their knee pain and symptoms, and that one or both interventions could eventually be implemented within the NHS.

For peer review only

Ethics and dissemination

This feasibility trial and current protocol (v5.0, 25/01/2022) was approved by the West of Scotland Research Ethics Committee 5 (REC5), reference number 20/WS/0006 prior to commencing recruitment, which is ongoing. The study is sponsored by the University of Leeds (Research Integrity and Governance), UK and is registered with the ISRCTN (Reference: 15564385; Supplement 3). All individuals assisting with the trial will be informed of any protocol amendments, which will be approved by the Sponsor before being submitted to the REC/HRA for approval. The results will be disseminated to the study grant funder, submitted for publication in peer-reviewed journals and where requested, a summary of the study findings will be disseminated to study participants. No study participants will be identifiable in the study results.

Data availability statement

The full protocol and statistical analysis plan will be available via the University of Leeds data repository. The full anonymised trial data set and statistical codes will be available 12 months following publication upon request.

For peer review only

For peer review only - http://bmjopen.bmj.com/site/about/guidelines.xhtml

Acknowledgments

The authors would like to thank Alex G Web Design, Melbourne, Australia for duplicating the My Knee Exercise website for use in the UK and SMS Solutions Australia for developing the SMS programme and online interface for use in the UK. The authors would like to thank the clinicians, physiotherapists, patients, PPI members and other experts who provided input into the refinement of the My Knee UK and Group E-Rehab programmes during Phase 1 of the study. The authors would also like to thank the LIRMM clinical trials staff for their help with administering this study.

Competing interests

None declared.

Author contributions

RSH, RKN and KLB co-developed the Australian SMS programme and My Knee Exercise website. Funding was secured by PGC (lead applicant) and GAM, SRK, EMAH, KLB and CC (co-applicants). PGC, SRK, KLB, CC, EMAH, GAM, MC and DGW designed this protocol. EMAH produced the statistical analysis plan. The UK exercise and SMS programmes were developed by PGC, CC, DGW and MC, DGW respectively. DGW designed the UK version of the website (My Knee UK), created the Microsoft Sway education package, is leading the coordination of the study, and drafted the manuscript. All authors contributed to manuscript reviewing and editing and approved the final version.

Funding

This work is being supported by a grant from Versus Arthritis (grant number 22091). This paper presents independent research funded/supported by the National Institute for Health Research (NIHR) Leeds Biomedical Research Centre (BRC). The views expressed are those of the authors and

For peer review only

For peer review only - http://bmjopen.bmj.com/site/about/guidelines.xhtml

not necessarily those of the NIHR or the Department of Health and Social Care. RSH is supported by a National Health and Medical Research Council Senior Research Fellowship (#1154217). The funding sources and sponsor had no role in the study design, writing the manuscript or the decision to submit for publication.

For peer review only

REFERENCES

1 Thomas E, Peat G, Mallen C, *et al.* Predicting the course of functional limitation among older adults with knee pain: Do local signs, symptoms and radiographs add anything to general indicators? *Ann Rheum Dis* 2008;67:1390–8. doi:10.1136/ard.2007.080945

2 Jinks C, Jordan K, Croft P. Measuring the population impact of knee pain and disability with the Western Ontario and McMaster Universities Osteoarthritis Index (WOMAC). *Pain* 2002;100:55–64. doi:10.1016/S0304-3959(02)00239-7

3 Cui A, Li H, Wang D, *et al.* Global, regional prevalence, incidence and risk factors of knee osteoarthritis in population-based studies. *EClinicalMedicine* 2020;29–30. doi:10.1016/j.eclinm.2020.100587

4 Swain S, Sarmanova A, Mallen C, *et al.* Trends in incidence and prevalence of osteoarthritis in the United Kingdom: findings from the Clinical Practice Research Datalink (CPRD). *Osteoarthr Cartil* 2020;28:792–801. doi:10.1016/j.joca.2020.03.004

5 Arthritis Research UK. Osteoarthritis in General Practice: Data and perspectives. 2013. https://www.versusarthritis.org/media/2115/osteoarthritis-in-general-practice.pdf

6 Conaghan PG, Porcheret M, Kingsbury SR, *et al.* Impact and therapy of osteoarthritis: the Arthritis Care OA Nation 2012 survey. *Clin Rheumatol* 2015;34:1581–8. doi:10.1007/s10067-014-2692-1

7 NICE. Osteoarthritis : care and management (Clinical guideline [CG177]). 2014. https://www.nice.org.uk/guidance/cg177

8 Lawford BJ, Bennell KL, Kasza J, *et al.* Physical Therapists' Perceptions of Telephone- and Internet Video–Mediated Service Models for Exercise Management of People With Osteoarthritis. *Arthritis Care Res* 2018;70:398–408. doi:10.1002/acr.23260

9 Bennell KL, Nelligan R, Dobson F, *et al.* Effectiveness of an internet-delivered exercise and pain-coping skills training intervention for persons with chronic knee pain: A randomized trial. *Ann Intern Med* 2017;166:453–62. doi:10.7326/M16-1714

10 Bennell KL, Lawford BJ, Keating C, *et al.* Comparing Video-Based, Telehealth-Delivered Exercise and Weight Loss Programs With Online Education on Outcomes of Knee Osteoarthritis: A Randomized Trial. *Ann Intern Med* Published Online First: 2021. doi:10.7326/M21-2388

For peer review only

11 NHS England and NHS Improvement. Clinical guide for the management of remote consultations and remote working in secondary care during the coronavirus pandemic. *NHS Engl* 2020;:1–16.https://www.nice.org.uk/media/default/about/covid-19/specialty-guides/specialty-guide-virtual-working-and-coronavirus.pdf

12 Vusirikala A, Ensor D, Asokan AK, *et al.* Hello, can you hear me? Orthopaedic clinic telephone consultations in the COVID-19 era- a patient and clinician perspective. *World J Orthop* 2021;12:24–34. doi:10.5312/WJO.V12.I1.24

13 Bennell KL, Lawford BJ, Metcalf B, *et al.* Physiotherapists and patients report positive experiences overall with telehealth during the COVID-19 pandemic: a mixed-methods study. *J Physiother* 2021;67:201–9. doi:10.1016/j.jphys.2021.06.009

14 Age UK. Digital inclusion and older people – how have things changed in a Covid-19 world? London: 2021. https://www.ageuk.org.uk/globalassets/age-uk/documents/reports-and-publications/reports-and-briefings/active-communities/digital-inclusion-in-the-pandemic-final-march-2021.pdf (accessed 4 Nov 2021).

15 Nelligan RK, Hinman RS, Kasza J, *et al.* Effects of a Self-directed Web-Based Strengthening Exercise and Physical Activity Program Supported by Automated Text Messages for People with Knee Osteoarthritis: A Randomized Clinical Trial. *JAMA Intern Med* 2021;181:776–85. doi:10.1001/jamainternmed.2021.0991

16 Nelligan RK, Hinman RS, Teo PL, *et al.* Exploring attitudes and experiences of people with knee osteoarthritis toward a self-directed ehealth intervention to support exercise: Qualitative Study. *JMIR Rehabil Assist Technol* 2020;7. doi:10.2196/18860

17 Horton M. CSP: Cuts to physiotherapy services a false economy. Just Physio. 2016;:Online.https://www.justphysio.co.uk/physiotherapy-article-801817162-csp_cuts_to_physiotherapy.html (accessed 22 Jul 2021).

18 Oxtoby K. Covid-19: "Life on hold" for NHS patients needing musculoskeletal care. *BMJ* 2021;373:n1616. doi:10.1136/bmj.n1616

19 Paul J, Hanna JB. Applying the marketing concept in health care: The no-show problem. *Health Mark Q* 1997;14:3–17. doi:10.1300/J026v14n03_02

20 Tan E, Shah A, De Souza W, *et al.* Improving the patient booking service to reduce the number of missed appointments at East London NHS Foundation Trust Community Musculoskeletal Physiotherapy Service. *BMJ Open Qual* 2017;6:e000093. doi:10.1136/BMJOQ-2017-000093

For peer review only

For peer review only - http://bmjopen.bmj.com/site/about/guidelines.xhtml

21 Collins J, Santamaria N, Clayton L. Why outpatients fail to attend their scheduled appointments: a prospective comparison of differences between attenders and non-attenders. *Aust Health Rev* 2003;26:52–63. doi:10.1071/AH030052

22 Chan A-W, Tetzlaff JM, Gøtzsche PC, *et al.* SPIRIT 2013 explanation and elaboration: guidance for protocols of clinical trials. *BMJ* 2013;346. doi:10.1136/bmj.e7586

23 Chan A-W, Tetzlaff JM, Altman DG, *et al.* SPIRIT 2013 Statement: Defining Standard Protocol Items for Clinical Trials. *Ann Intern Med* 2013;158:200–7. doi:10.7326/0003-4819-158-3-201302050-00583

24 Eldridge SM, Chan CL, Campbell MJ, *et al.* CONSORT 2010 statement: Extension to randomised pilot and feasibility trials. *Pilot Feasibility Stud* 2016;2. doi:10.1186/s40814-016-0105-8

25 Orkin AM, Gill PJ, Ghersi D, *et al.* Guidelines for Reporting Trial Protocols and Completed Trials Modified Due to the COVID-19 Pandemic and Other Extenuating Circumstances: The CONSERVE 2021 Statement. *JAMA* Published Online First: 21 June 2021. doi:10.1001/jama.2021.9941

26 Jensen MP, Karoly P. Self-report scales and procedures for assessing pain in adults. In: Turk DC, Melzack R, eds. *Handbook of Pain Assessment*. New York: : Guilford Press 2011. 19–44.https://web.s.ebscohost.com/ehost/ebookviewer/ebook/bmxlYmtfXzM0NjE5Ml9fQU41?sid=487b6ed8-a929-49e5-8236-72b3dc113ff7@redis&vid=0&format=EB&lpid=lp_3&rid=0

27 Nelligan RK, Hinman RS, McManus F, *et al.* Moderators of the Effect of a Self-directed Digitally Delivered Exercise Program for People With Knee Osteoarthritis: Exploratory Analysis of a Randomized Controlled Trial. *J Med Internet Res* 2021;23:e30768. doi:10.2196/30768

28 Hurley M, Dickson K, Hallett R, *et al.* Exercise interventions and patient beliefs for people with hip, knee or hip and knee osteoarthritis: A mixed methods review. *Cochrane Database Syst Rev* Published Online First: 2018. doi:10.1002/14651858.CD010842.pub2

29 Abroms LC, Whittaker R, Free C, *et al.* Developing and pretesting a text messaging program for health behavior change: Recommended steps. *JMIR mHealth uHealth* 2015;3. doi:10.2196/mhealth.4917

30 Bennell K, Nelligan RK, Schwartz S, *et al.* Behavior change text messages for home exercise adherence in knee osteoarthritis: Randomized trial. *J Med Internet Res* 2020;22:e21749. doi:10.2196/21749

For peer review only

31 Nelligan RK, Hinman RS, Atkins L, *et al.* A short message service intervention to support adherence to home-based strengthening exercise for people with knee osteoarthritis: Intervention design applying the behavior change wheel. *JMIR mHealth uHealth* 2019;7:1–12. doi:10.2196/14619

32 Jones CJ, Rikli RE, Beam WC. A 30-s chair-stand test as a measure of lower body strength in community-residing older adults. *Res Q Exerc Sport* 1999;70:113–9. doi:10.1080/02701367.1999.10608028

33 Microsoft Corporation. Microsoft Sway [Internet]. 2021.https://sway.office.com (accessed 1 Nov 2021).

34 Sealed Envelope Ltd. Create a blocked randomisation list. 2021.https://www.sealedenvelope.com/simple-randomiser/v1/lists (accessed 18 Jan 2021).

35 Strand V, Kelman A. Outcome measures in osteoarthritis: randomized controlled trials. Curr. Rheumatol. Rep. 2004;6:20–30. doi:10.1007/s11926-004-0080-6

36 Sharma A, Kudesia P, Shi Q, *et al.* Anxiety and depression in patients with osteoarthritis: Impact and management challenges. *Open Access Rheumatol Res Rev* 2016;8:103–13. doi:10.2147/OARRR.S93516

37 McAlindon TE, Driban JB, Henrotin Y, *et al.* OARSI Clinical Trials Recommendations: Design, conduct, and reporting of clinical trials for knee osteoarthritis. *Osteoarthr Cartil* 2015;23:747–60. doi:10.1016/j.joca.2015.03.005

38 Jackson T, Xu T, Jia X. Arthritis self-efficacy beliefs and functioning among osteoarthritis and rheumatoid arthritis patients: A meta-analytic review. *Rheumatol (United Kingdom)* 2020;59:948–58. doi:10.1093/rheumatology/kez219

39 Google.com. About Google Analytics. 2021.https://marketingplatform.google.com/intl/en_uk/about/analytics/ (accessed 1 Nov 2021).

40 Lancaster G, Lancaster GA, Dodd S, *et al.* Correspondence Design and analysis of pilot studies: recommendations for good practice. *J Eval Clin Pract* 2004;10:307–12. doi:10.1111/j..2002.384.doc.x

41 Angst F, Aeschlimann A, Stucki G. Smallest Detectable and Minimal Clinically Important Differences of Rehabilitation Intervention With Their Implications for Required Sample Sizes

For peer review only

For peer review only - http://bmjopen.bmj.com/site/about/guidelines.xhtml

Using WOMAC and SF-36 Quality of Life Measurement Instruments in Patients With Osteoarthritis of the Lower Ex. *Arthritis Care Res (Hoboken)* 2001;45:384–91. doi:10.1002/1529-0131(200108)45:4<384::AID-ART352>3.0.CO;2-0

42 Lee EC, Whitehead AL, Jacques RM, *et al.* The statistical interpretation of pilot trials: Should significance thresholds be reconsidered? *BMC Med Res Methodol* 2014;14:1–8. doi:10.1186/1471-2288-14-41

43 Ritchie J, Spencer L. Qualitative data analysis for applied policy research. In: Bryman A, Burgess RG, eds. *Analyzing Qalitative Data*. London: : Routledge 1994. 173–94. doi:10.4324/9780203413081_chapter_9

44 Choi W, Zheng H, Franklin P, *et al.* mHealth technologies for osteoarthritis self-management and treatment: A systematic review. *Health Informatics J* 2019;25:984–1003. doi:10.1177/1460458217735676

45 Fransen M, Mcconnell S, Harmer AR, *et al.* Exercise for osteoarthritis of the knee. Cochrane Database Syst. Rev. 2015;2015. doi:10.1002/14651858.CD004376.pub3

For peer review only

For peer review only - http://bmjopen.bmj.com/site/about/guidelines.xhtml

TABLE AND FIGURE TITLES AND LEGENDS

Table 1: Trial eligibility criteria.

Figure 1: Study flow diagram.

Table 2: Summary of the My Knee UK rehabilitation programme.

Table 3: Summary of the Group E-Rehab rehabilitation programme.

Table 4: SPIRIT (Standard Protocol Items for Randomized Trials) schedule of enrolment, interventions, and assessments.

[NRS: numeric rating scale; WOMAC: The Western Ontario and McMaster Universities Osteoarthritis Index; ASES: Arthritis Self-Efficacy Scale; HADS: Hospital Anxiety and Depression Scale; EQ-5D-5L; European Quality of Life-Five Dimension-Five Level Scale; SF-12: 12-Item Short Form Survey]

SUPPLEMENTS

Supplement 1: Protocol modifications made to mitigate the effects of the Covid-19 Pandemic

Supplement 2: Sample participant consent form for the Phase 2 trial

Supplement 3: Trial registration

For peer review only

For peer review only - http://bmjopen.bmj.com/site/about/guidelines.xhtml

EXCLUSIVE LICENCE

I, the Submitting Author has the right to grant and does grant on behalf of all authors of the Work (as defined in the below author licence), an exclusive licence and/or a non-exclusive licence for contributions from authors who are: i) UK Crown employees; ii) where BMJ has agreed a CC-BY licence shall apply, and/or iii) in accordance with the terms applicable for US Federal Government officers or employees acting as part of their official duties; on a worldwide, perpetual, irrevocable, royalty-free basis to BMJ Publishing Group Ltd ("BMJ") its licensees and where the relevant Journal is co-owned by BMJ to the co-owners of the Journal, to publish the Work in BMJ Open and any other BMJ products and to exploit all rights, as set out in our licence.

The Submitting Author accepts and understands that any supply made under these terms is made by BMJ to the Submitting Author unless you are acting as an employee on behalf of your employer or a postgraduate student of an affiliated institution which is paying any applicable article publishing charge ("APC") for Open Access articles. Where the Submitting Author wishes to make the Work available on an Open Access basis (and intends to pay the relevant APC), the terms of reuse of such Open Access shall be governed by a Creative Commons licence – details of these licences and which Creative Commons licence will apply to this Work are set out in our licence referred to above.

For peer review only

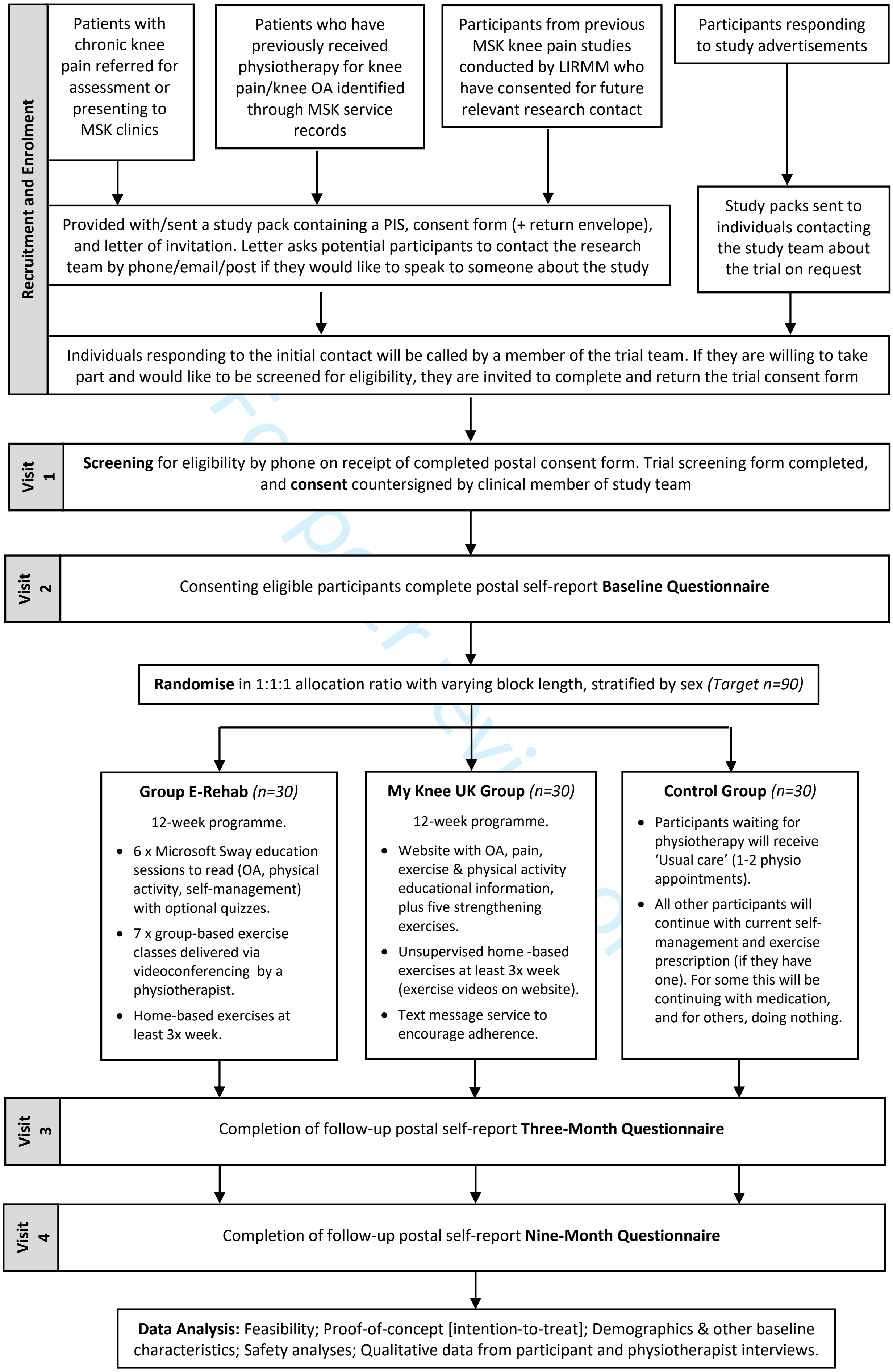

For peer review only - http://bmjopen.bmj.com/site/about/guidelines.xhtml

SUPPLEMENT 1

Protocol modifications made to mitigate the effects of the Covid-19 Pandemic

1. Changes arising from the Covid-19 pandemic mean that routine physiotherapy appointments are now more likely to be remote rather than face-to-face. Currently, most appointments are taking place over the telephone or in some case, via video consultation, rather than being face-to-face in a clinic. Prior to starting the recruitment process for the feasibility trial, the protocol and participant documents were amended to reflect these changes. This means that potential participants will be fully aware that if they enter the study and are allocated to the control group, if they are current patients who will receive usual care, then their physiotherapy appointments are unlikely to be face-to-face, particularly if Covid-19 restrictions are still in place.

2. Given the current and potential future restrictions arising from the Covid-19 pandemic, interviews with the physiotherapists and sub-sample of participants from the trial during the nested qualitative study phase will be done remotely.

3. Current Covid-19 restrictions mean that only skeleton staff members from the research team were physically going into the office. This would have caused a delay in responding to the initial postal response to enquiries from potential participants. To avoid this, the invitation letter was amended slightly so that if anyone receiving the study information is interested in joining the study, they are asked to contact the study team via email or phone instead of returning a paper contact form.

4. A delayed start due to Covid-19 has resulted in shortened timeframe for recruitment, with the risk that reaching the target of 90 participants within the study timeframe would not be achievable. To mitigate this, additional ways of identifying and recruiting participants were added to the protocol. This included:

For peer review only - http://bmjopen.bmj.com/site/about/guidelines.xhtml

- Searching local Trust MSK service records for potentially eligible patients who have previously received physio for knee pain.
- Using Participant identification Centres (PICs) who have the appropriate approval and model agreement in place to carrying out a search of their patient records database to identify individuals that meet a study's eligibility criteria.
- A database search of participants from previous University of Leeds studies who have consented to be contacted about future relevant research studies.
- A media campaign with physical posters/flyers in clinics, waiting rooms or local community centres, and an electronic poster/advert that can be distributed digitally (e.g. email, University or Trust websites), and used via social media.

Participants recruited via MSK waiting lists or clinics (current patients) will continue to receive a physio appointment (usual care) if they are randomised to the control group or if they subsequently withdraw. However, the amendment will affect participants identified via service records, database searches, media campaigns or PICs if they are randomised to the control group or later withdraw from the trial. Participants recruited using these methods (i.e. not via the MSK waiting list or clinics), will be asked to continue with whatever exercises, treatment, or self-management they were doing before joining the trial.

For peer review only

For peer review only - http://bmjopen.bmj.com/site/about/guidelines.xhtml

SUPPLEMENT 2

Sample participant consent form for the Phase 2 trial

UNIVERSITY OF LEEDS
Leeds Institute of Rheumatic and Musculoskeletal Medicine
2nd Floor, Chapel Allerton Hospital
Chapeltown Road, Leeds, LS7 4SA
Email: e.rehab@leeds.ac.uk
Tel: 0113 392 4965

Participant Identification Number [ ]

CONSENT FORM (IRAS Reference: 269827)

Evaluation of electronic-rehabilitation programmes for chronic knee pain

Phase 2: Feasibility trial

Please initial each box

1	I confirm that I have read the information sheet dated 18/05/2021 (version 3.0) for the above study. I have had the opportunity to consider the information, ask questions and have had these answered satisfactorily.	
2	I understand that my participation is voluntary and that I am free to withdraw at any time without giving any reason, and without my legal rights or medical care being affected.	
3	I understand that, even if I withdraw from this study, the information already collected from me will be used in analysing the results of this study.	
4	I understand that the group sessions will be recorded and any identifiable audio and visual data from these recordings will be stored securely and not shared.	
5	I understand that the information collected during this study may be used to support other research in the future and may be shared anonymously with other researchers.	
6	I agree to my personal information being stored for the purposes of this study. I understand that any information which could identify me will be kept strictly confidential.	
7	I understand that relevant sections of the data collected during the study, may be looked at by individuals from the University of Leeds (sponsors) or from regulatory authorities where it is relevant to my taking part in this research.	
8	I understand that my GP will be informed about my participation in this study.	
9	I agree to take part in the above study.	

After initialling the boxes, please print and sign your name (participant), add the date, and fill out the participant contact details on the next page before returning the form.

For peer review only - http://bmjopen.bmj.com/site/about/guidelines.xhtml

______________________________ ______________ ________________________

Name of Participant (PRINTED) Date Signature

Please remember to fill in your contact details at the bottom of the page.

The contact slip will be removed and destroyed after we have put your details on to our secure Subject Screening Log, which is used by the research team for contacting you about the study.

______________________________ ______________ ________________________

Name of Researcher *Date Signature

When completed: 1 for participant; 1 for researcher site file. *Will be signed by researcher on a different day.

✂...

<u>Participant Contact Details</u>

Telephone Number(s): ____

Email Address: ____

IRAS ID	*Project Title*	*Document Type*	*Version #*	*Date*	*Page*
269827	E-rehab for Knee Pain	Phase 2 Trial Patient Consent Form	4.0	10/09/2021	**Page 2 of 2**

For peer review only - http://bmjopen.bmj.com/site/about/guidelines.xhtml

For peer review only

SUPPLEMENT 3

Trial registration

Data category	Information
Registry identifying number	ISRCTN15564385
Date of registration	07/02/2020
Prospective/Retrospective	Prospectively registered
Additional identifiers	CPMS 43473, IRAS 269827
Sources of monetary support	Versus Arthritis; National Institute for Health Research (NIHR) (UK)
Sponsor	University of Leeds
Contact information	Dr Dawn Groves-Williams; d.groves-williams@leeds.ac.uk
	Dr Sarah Kingsbury; s.r.kingsbury@leeds.ac.uk
Short/public title	E-rehab for Knee Pain
Scientific title	Evaluation of electronic-rehabilitation programmes for chronic knee pain
Countries of recruitment	United Kingdom
Condition category	Musculoskeletal Diseases
Condition	Chronic knee pain
Interventions	Access to **My Knee UK** website containing exercise videos and self-management resources for 12 weeks. Support via SMS messages
	**Group E-Rehab**: access to online educational and self-management resources plus 7 online group physiotherapy classes over 12-weeks
	**Control Group**: depending on their recruitment path – will receive usual physiotherapy care or continue with usual self-management
Key inclusion and exclusion criteria	Type: Adults ≥ 45 years; both sexes; no healthy volunteers
	Inclusion: knee pain > 3 months and on most days of previous month; activity-related joint pain; pain during walking ≥4 on an 11-point scale
	Exclusion: Inflammatory arthritis/gout; Joint replacement in study knee; injection within last month; arthroscopy within last 3-months
Study design	Mixed methods interventional randomised controlled feasibility trial with follow-up interviews
	Primary: Interventional; Secondary: Randomised controlled trial; Trial setting: Community; Trial type: treatment
Target sample size	90 (30 in each group)
Date of first enrolment	22/04/2021
Recruitment status	Recruiting
Overall trial status	Ongoing
Key outcomes (Primary and secondary outcomes have not been specified due to this being a feasibility study).	Participants and physiotherapists find e-rehabilitation feasible and acceptable, and it can be administered successfully; calculation of a sample size that can be achieved in a main trial; max. of 30% attrition at 3-months; ≥ 40-50% of eligible participants are recruited.
	Patient-reported outcome domains are: pain; quality of life; pain coping and catastrophising; resource use; confidence and motivation to do exercises; global change in overall pain and mobility/function.

For peer review only - http://bmjopen.bmj.com/site/about/guidelines.xhtml

Reporting checklist for protocol of a clinical trial.

Based on the SPIRIT guidelines.

Instructions to authors

Complete this checklist by entering the page numbers from your manuscript where readers will find each of the items listed below.

Your article may not currently address all the items on the checklist. Please modify your text to include the missing information. If you are certain that an item does not apply, please write "n/a" and provide a short explanation.

Upload your completed checklist as an extra file when you submit to a journal.

In your methods section, say that you used the SPIRITreporting guidelines, and cite them as:

Chan A-W, Tetzlaff JM, Gøtzsche PC, Altman DG, Mann H, Berlin J, Dickersin K, Hróbjartsson A, Schulz KF, Parulekar WR, Krleža-Jerić K, Laupacis A, Moher D. SPIRIT 2013 Explanation and Elaboration: Guidance for protocols of clinical trials. BMJ. 2013;346:e7586

		Reporting Item	Page Number
**Administrative information**			
Title	#1	Descriptive title identifying the study design, population, interventions, and, if applicable, trial acronym	1
Trial registration	#2a	Trial identifier and registry name. If not yet registered, name of intended registry	4
Trial registration: data set	#2b	All items from the World Health Organization Trial Registration Data Set	Suppl 3
Protocol version	#3	Date and version identifier	21
Funding	#4	Sources and types of financial, material, and other support	23-24
Roles and responsibilities: contributorship	#5a	Names, affiliations, and roles of protocol contributors	1,23

For peer review only

Roles and responsibilities: sponsor contact information	#5b	Name and contact information for the trial sponsor	22
Roles and responsibilities: sponsor and funder	#5c	Role of study sponsor and funders, if any, in study design; collection, management, analysis, and interpretation of data; writing of the report; and the decision to submit the report for publication, including whether they will have ultimate authority over any of these activities	24
Roles and responsibilities: committees	#5d	Composition, roles, and responsibilities of the coordinating centre, steering committee, endpoint adjudication committee, data management team, and other individuals or groups overseeing the trial, if applicable (see Item 21a for data monitoring committee)	8,23
**Introduction**			
Background and rationale	#6a	Description of research question and justification for undertaking the trial, including summary of relevant studies (published and unpublished) examining benefits and harms for each intervention	6-7
Background and rationale: choice of comparators	#6b	Explanation for choice of comparators	7
Objectives	#7	Specific objectives or hypotheses	7
Trial design	#8	Description of trial design including type of trial (eg, parallel group, crossover, factorial, single group), allocation ratio, and framework (eg, superiority, equivalence, non-inferiority, exploratory)	3,7
**Methods: Participants, interventions, and outcomes**			
Study setting	#9	Description of study settings (eg, community clinic, academic hospital) and list of countries where data will	Fg1

For peer review only

		be collected. Reference to where list of study sites can be obtained	
Eligibility criteria	#10	Inclusion and exclusion criteria for participants. If applicable, eligibility criteria for study centres and individuals who will perform the interventions (eg, surgeons, psychotherapists)	Fg1
Interventions: description	#11a	Interventions for each group with sufficient detail to allow replication, including how and when they will be administered	9-15
Interventions: modifications	#11b	Criteria for discontinuing or modifying allocated interventions for a given trial participant (eg, drug dose change in response to harms, participant request, or improving / worsening disease)	N/A
Interventions: adherance	#11c	Strategies to improve adherence to intervention protocols, and any procedures for monitoring adherence (eg, drug tablet return; laboratory tests)	9,12
Interventions: concomitant care	#11d	Relevant concomitant care and interventions that are permitted or prohibited during the trial	8(Tbl1)
Outcomes	#12	Primary, secondary, and other outcomes, including the specific measurement variable (eg, systolic blood pressure), analysis metric (eg, change from baseline, final value, time to event), method of aggregation (eg, median, proportion), and time point for each outcome. Explanation of the clinical relevance of chosen efficacy and harm outcomes is strongly recommended	16-19
Participant timeline	#13	Time schedule of enrolment, interventions (including any run-ins and washouts), assessments, and visits for participants. A schematic diagram is highly recommended (see Figure)	7, 17(Tbl4)
Sample size	#14	Estimated number of participants needed to achieve study objectives and how it was determined, including clinical and statistical assumptions supporting any sample size calculations	19
Recruitment	#15	Strategies for achieving adequate participant enrolment to reach target sample size	8, Fig1

For peer review only

**Methods: Assignment of interventions (for controlled trials)**			
Allocation: sequence generation	#16a	Method of generating the allocation sequence (eg, computer-generated random numbers), and list of any factors for stratification. To reduce predictability of a random sequence, details of any planned restriction (eg, blocking) should be provided in a separate document that is unavailable to those who enrol participants or assign interventions	15
Allocation concealment mechanism	#16b	Mechanism of implementing the allocation sequence (eg, central telephone; sequentially numbered, opaque, sealed envelopes), describing any steps to conceal the sequence until interventions are assigned	15
Allocation: implementation	#16c	Who will generate the allocation sequence, who will enrol participants, and who will assign participants to interventions	15
Blinding (masking)	#17a	Who will be blinded after assignment to interventions (eg, trial participants, care providers, outcome assessors, data analysts), and how	15
Blinding (masking): emergency unblinding	#17b	If blinded, circumstances under which unblinding is permissible, and procedure for revealing a participant's allocated intervention during the trial	N/A
**Methods: Data collection, management, and analysis**			
Data collection plan	#18a	Plans for assessment and collection of outcome, baseline, and other trial data, including any related processes to promote data quality (eg, duplicate measurements, training of assessors) and a description of study instruments (eg, questionnaires, laboratory tests) along with their reliability and validity, if known.	16

For peer review only

		Reference to where data collection forms can be found, if not in the protocol	
Data collection plan: retention	#18b	Plans to promote participant retention and complete follow-up, including list of any outcome data to be collected for participants who discontinue or deviate from intervention protocols	16
Data management	#19	Plans for data entry, coding, security, and storage, including any related processes to promote data quality (eg, double data entry; range checks for data values). Reference to where details of data management procedures can be found, if not in the protocol	16
Statistics: outcomes	#20a	Statistical methods for analysing primary and secondary outcomes. Reference to where other details of the statistical analysis plan can be found, if not in the protocol	19-21
Statistics: additional analyses	#20b	Methods for any additional analyses (eg, subgroup and adjusted analyses)	3,18
Statistics: analysis population and missing data	#20c	Definition of analysis population relating to protocol non-adherence (eg, as randomised analysis), and any statistical methods to handle missing data (eg, multiple imputation)	19
**Methods: Monitoring**			
Data monitoring: formal committee	#21a	Composition of data monitoring committee (DMC); summary of its role and reporting structure; statement of whether it is independent from the sponsor and competing interests; and reference to where further details about its charter can be found, if not in the protocol. Alternatively, an explanation of why a DMC is not needed	N/A (non-CTIMP)
Data monitoring: interim analysis	#21b	Description of any interim analyses and stopping guidelines, including who will have access to these interim results and make the final decision to terminate the trial	N/A
Harms	#22	Plans for collecting, assessing, reporting, and managing solicited and spontaneously reported adverse events	20

For peer review only

		and other unintended effects of trial interventions or trial conduct	
Auditing	#23	Frequency and procedures for auditing trial conduct, if any, and whether the process will be independent from investigators and the sponsor	16
**Ethics and dissemination**			
Research ethics approval	#24	Plans for seeking research ethics committee / institutional review board (REC / IRB) approval	4,22
Protocol amendments	#25	Plans for communicating important protocol modifications (eg, changes to eligibility criteria, outcomes, analyses) to relevant parties (eg, investigators, REC / IRBs, trial participants, trial registries, journals, regulators)	21-22
Consent or assent	#26a	Who will obtain informed consent or assent from potential trial participants or authorised surrogates, and how (see Item 32)	15
Consent or assent: ancillary studies	#26b	Additional consent provisions for collection and use of participant data and biological specimens in ancillary studies, if applicable	N/A
Confidentiality	#27	How personal information about potential and enrolled participants will be collected, shared, and maintained in order to protect confidentiality before, during, and after the trial	16,22
Declaration of interests	#28	Financial and other competing interests for principal investigators for the overall trial and each study site	22
Data access	#29	Statement of who will have access to the final trial dataset, and disclosure of contractual agreements that limit such access for investigators	22
Ancillary and post trial care	#30	Provisions, if any, for ancillary and post-trial care, and for compensation to those who suffer harm from trial participation	N/A
Dissemination policy: trial results	#31a	Plans for investigators and sponsor to communicate trial results to participants, healthcare professionals, the	4, 22

For peer review only

		public, and other relevant groups (eg, via publication, reporting in results databases, or other data sharing arrangements), including any publication restrictions	
Dissemination policy: authorship	#31b	Authorship eligibility guidelines and any intended use of professional writers	31
Dissemination policy: reproducible research	#31c	Plans, if any, for granting public access to the full protocol, participant-level dataset, and statistical code	22
**Appendices**			
Informed consent materials	#32	Model consent form and other related documentation given to participants and authorised surrogates	Suppl 2
Biological specimens	#33	Plans for collection, laboratory evaluation, and storage of biological specimens for genetic or molecular analysis in the current trial and for future use in ancillary studies, if applicable	N/A

None The SPIRIT Explanation and Elaboration paper is distributed under the terms of the Creative Commons Attribution License CC-BY-NC. This checklist can be completed online using https://www.goodreports.org/, a tool made by the EQUATOR Network in collaboration with Penelope.ai

For peer review only
