# Supplementary material for: Evaluation of two electronic-rehabilitation programmes for persistent knee pain: protocol for a randomised feasibility trial
Source: BMJ Open. 2022 Jun 3;12(6):e063608. doi: 10.1136/bmjopen-2022-063608 (PMC9171213; doi:10.1136/bmjopen-2022-063608)
Supplement: Reviewer comments [file bmjopen-2022-063608.reviewer_comments.pdf]

PEER REVIEW HISTORY

BMJ Open publishes all reviews undertaken for accepted manuscripts. Reviewers are asked to complete a checklist review form (**http://bmjopen.bmj.com/site/about/resources/checklist.pdf**) and are provided with free text boxes to elaborate on their assessment. These free text comments are reproduced below.

ARTICLE DETAILS

**TITLE (PROVISIONAL)**	Evaluation of two electronic-rehabilitation programmes for persistent knee pain: protocol for a randomised feasibility trial
**AUTHORS**	Groves-Williams, Dawn; McHugh, Gretl; Bennell, Kim; Comer, Christine; Hensor, Liz; Conner, Mark; Nelligan, Rachel; Hinman, Rana; Kingsbury, Sarah; Conaghan, Philip

This article was not externally reviewed at BMJ Open. Protocols that have been independently assessed prior to submission to BMJ Open are usually fast-tracked to publication on the grounds that further substantial changes will not be possible. This independent assessment will usually be external, independent review for both a substantial grant award from a non-commercial or government funder and ethics approval.
